# A meta-regression analysis of 41 Australian problem gambling prevalence estimates and their relationship to total spending on electronic gaming machines

**DOI:** 10.1186/s12889-017-4413-6

**Published:** 2017-05-23

**Authors:** Francis Markham, Martin Young, Bruce Doran, Mark Sugden

**Affiliations:** 1 Fenner School of Environment and Society, The Australian National University, 48A Linnaeus Way, Acton, ACT 2601 Australia; 2 School of Business and Tourism, Southern Cross University, Hogbin Drive, Coffs Harbour, NSW 2450 Australia

**Keywords:** Gambling, Total consumption theory, Problem gambling, Problem gambling prevalence, Electronic gaming machines, Moderate-risk problem gambling, Gambling expenditure, Gambling losses

## Abstract

**Background:**

Many jurisdictions regularly conduct surveys to estimate the prevalence of problem gambling in their adult populations. However, the comparison of such estimates is problematic due to methodological variations between studies. Total consumption theory suggests that an association between mean electronic gaming machine (EGM) and casino gambling losses and problem gambling prevalence estimates may exist. If this is the case, then changes in EGM losses may be used as a proxy indicator for changes in problem gambling prevalence. To test for this association this study examines the relationship between aggregated losses on electronic gaming machines (EGMs) and problem gambling prevalence estimates for Australian states and territories between 1994 and 2016.

**Methods:**

A Bayesian meta-regression analysis of 41 cross-sectional problem gambling prevalence estimates was undertaken using EGM gambling losses, year of survey and methodological variations as predictor variables. General population studies of adults in Australian states and territory published before 1 July 2016 were considered in scope. 41 studies were identified, with a total of 267,367 participants. Problem gambling prevalence, moderate-risk problem gambling prevalence, problem gambling screen, administration mode and frequency threshold were extracted from surveys. Administrative data on EGM and casino gambling loss data were extracted from government reports and expressed as the proportion of household disposable income lost.

**Results:**

Money lost on EGMs is correlated with problem gambling prevalence. An increase of 1% of household disposable income lost on EGMs and in casinos was associated with problem gambling prevalence estimates that were 1.33 times higher [95% credible interval 1.04, 1.71]. There was no clear association between EGM losses and moderate-risk problem gambling prevalence estimates. Moderate-risk problem gambling prevalence estimates were not explained by the models (*I* ^2^ ≥ 0.97; *R* ^2^ ≤ 0.01).

**Conclusions:**

The present study adds to the weight of evidence that EGM losses are associated with the prevalence of problem gambling. No patterns were evident among moderate-risk problem gambling prevalence estimates, suggesting that this measure is either subject to pronounced measurement error or lacks construct validity. The high degree of residual heterogeneity raises questions about the validity of comparing problem gambling prevalence estimates, even after adjusting for methodological variations between studies.

**Electronic supplementary material:**

The online version of this article (doi:10.1186/s12889-017-4413-6) contains supplementary material, which is available to authorized users.

## Background

### Introduction and rationale

Total consumption theory, or single distribution theory as it is sometimes known, predicts that the incidence of gambling-related harm is related to the amount of time and money spent on gambling within a given jurisdiction [1, 2]. This prediction derives from a postulate of total consumption theory that, at the population level, a fixed proportion of total gambling activity will result in harm. Any growth in gambling will accordingly produce a proportionate growth in harm. If this hypothesis is correct, it implies that gambling-related harm would be best prevented by reducing the gambling consumption of the entire population, not just those gambling to excess. A related implication is that changes in mean gambling consumption may be used as a proxy indicator for changes in problem gambling prevalence.

A small research literature has investigated the veracity of the propositions of total consumption theory as they relate to gambling. In a pioneering analysis of household expenditure surveys, Grun and McKeigue [3] found that mean gambling losses in British geographic regions were strongly correlated with the proportion of the population losing an ‘excessive’ sum of money on gambling, both before and after the introduction of the National Lottery. Lund [2], analysing three independent Norwegian samples, found similar correlations between average gambling frequency and the proportion of the population gambling very frequently.

These studies did not specifically examine gambling-related harms, a shortcoming addressed by both Hansen and Rossow [4] and Markham et al. [5]. Hansen and Rossow examined problem gambling among Norwegian adolescents grouped by school, and found a strong correlation among schools between average losses on slot machines and the reported prevalence of problem-gambling symptoms. Similarly, Markham et al. found that the reported prevalence of two or more problem-gambling symptoms among gamblers using electronic gaming machines (EGMs) in Australian venues was correlated with the average amount lost on EGMs in those venues.

Few studies, however, have systematically examined the relationship between gambling-related harm and gambling losses at the spatial scale of the regulatory jurisdiction (e.g. the country, state, territory, province, Bundesland, etc.). The jurisdictional spatial scale is important since jurisdictions compromise the territorial unit at which gambling is most frequently regulated, gambling losses are usually reported e.g. [6, 7], and problem gambling surveys are usually conducted [8]. In one notable example of a jurisdictional-level study, the Productivity Commission [9] surveyed problem gambling prevalence in all Australian states and territories and compared these prevalence estimates to total non-lottery gambling losses in the same jurisdictions, finding a positive correlation. Unfortunately, this study was constrained by design to an examination of only eight prevalence estimates, limiting its generalizability.

Counter-examples to predictions of total consumption theory have been forwarded by Abbott [10], who describes the reduction in problem gambling prevalence estimates in New Zealand over a 9 years period during which total gambling losses increased substantially. Consequently, in a series of studies [10–13], Abbott proposed an alternative hypothesis of ‘adaptation’, in which the prevalence of problem gambling tends to fall over time. The reasons for adaptation may include a decline in gambling participation as the novelty of a new gambling activity dwindles, decreased average duration of gambling problems through destigmatisation and improved treatment, changing cultural norms, increased knowledge of gambling-related harms, and the introduction of regulations such as in-venue smoking bans and caps on EGM numbers.

One explanation for the dearth of convincing evidence about the relationships between gambling harms and gambling losses at jurisdictional scales is the problem of inter-study heterogeneity, generally thought to result from a lack of methodological consistency between prevalence studies. Inter-jurisdictional comparisons may be compromised because problem gambling prevalence studies tend to use heterogeneous methods that limit comparability [8, 14–17]. As Sassen et al. [15] found in their systematic review of 39 studies, the decade between 2000 and 2010 saw little methodological convergence among prevalence studies. In practice, measurement differences between prevalence studies may be so great as to render comparisons between them invalid. Nevertheless, as an examination of almost any government-commissioned problem gambling prevalence study will demonstrate, comparisons between prevalence estimates are routinely drawn. Despite concerns regarding validity, almost every problem gambling prevalence study seeks to benchmark prevalence estimates against those within the same jurisdiction at a previous point in time, or within other jurisdictions at a similar point in time.

The problems inherent in comparing prevalence estimates have been recognised by some scholars, who have attempted to regularise these prevalence rates to account for methodological variations, e.g. [8, 13, 18, 19]. However, the validity of comparing regularised estimates has not yet been established because the amount of residual heterogeneity among studies *after* adjustment is unknown. This is important because problem gambling prevalence estimates are the primary means through which gambling-related harm is monitored by regulators and governments. If prevalence estimates cannot be meaningfully compared, this calls into question validity of the current, routine practice of monitoring problem gambling prevalence using surveys [16].

If the total consumption theory of gambling is correct, then monitoring total gambling losses might provide an alternative means to track the changing incidence of gambling-related harm. If gambling losses present an accurate and precise proxy measure for problem gambling prevalence, then the necessity to routinely conduct problem gambling prevalence estimates to monitor population-level rates of harm might be reduced. Instead, population-level gambling losses could be monitored as a proxy indicator for the incidence of harm in the population.

### Objectives

This study analyses the association between problem gambling prevalence estimates and gambling losses for Australian states and territories between 1994 and 2015. It aims to answer the following specific research questions:

- Is there an association between EGM and casino gambling losses and problem gambling prevalence estimates in Australian states and territories?
- What degree of heterogeneity remains in estimates of problem gambling prevalence after regularising for methodological variations, EGM gambling losses and year of survey?

## Methods

A meta-regression approach was used to estimate the association between EGM and casino gambling losses and problem gambling prevalence estimates for Australian states and territories.

### Setting

The units of analysis for this study were the eight states and territories of Australia. EGMs were introduced to these jurisdictions in a staggered manner, with New South Wales the first to legalise EGMs in 1956 [20]. This was followed by a wave of legalisations, mostly in the 1990s, which left Western Australia as the only jurisdiction without EGMs by 1997. Other legal gambling commodities that are available in all jurisdictions include lotteries, casino table games, instant lotteries, scratch cards, and betting on races, sports and special events.

Australia was selected as a study site because its eight federal states and territories pioneered the routine conduct of problem gambling prevalence studies, alongside Canada and the United States [8]. Consequently, there have been sufficient problem gambling prevalence studies conducted in Australia to warrant a meta-analysis of their results. The study was limited to Australian states and territories rather than including jurisdictions in multiple countries to minimise the potential differences among the populations surveyed.

### Data

Two sets of data were required:

1. problem gambling prevalence estimates and the characteristics of the studies which produced these estimates, and
2. EGM gambling losses in the state or territory that temporally match each prevalence study.

Problem gambling prevalence studies for Australian states and territories were identified through a systematic search process. Prevalence studies are most frequently published as reports in the ‘grey literature’ rather than as peer-reviewed journal articles. Consequently, the search strategy primarily involved the identification of this relatively well-known corpus of problem gambling prevalence studies from previous inventories [8, 21]. The websites of Australian government bodies that have commissioned problem gambling prevalence studies were searched to identify further studies for examination, as were the reference lists of identified studies. The search revealed one study for which the full text was unavailable. The lead author of this study was contacted by email and conducted data extraction at the current authors’ request. To be eligible for inclusion, prevalence studies had to: 1) target the general population aged 18 years or older, 2) measure 12- or 6-months problem gambling using a validated problem gambling screen, 3) report results for one or more whole states or territories in Australia, 4) have been published prior to 1 July 2016, and 5) report on independent samples rather than longitudinal studies measuring change among the same respondents over time. Data were extracted independently by two coders, MS and FM. In cases where these two coders disagreed, data were coded independently by a third coder MY and a consensus meeting held, as discussed by Orwin and Vevea [22].

EGM gambling losses were selected as the predictor variable of interest in preference to total gambling losses because expenditure on different forms of gambling produces differing levels of harm. Previous research has shown that EGM and casino losses are more closely associated with problem gambling than either total gambling losses or losses on other gambling products [23, 24]. Gambling losses on casino table games were also included in the study as Australian player loss statistics for casinos do not distinguish between EGM and non-EGM gambling losses [7]. Gambling losses on EGMs during the year of survey fieldwork were extracted from *Australian Gambling Statistics, 32nd Edition*, a complete and authoritative administrative dataset [7]. This dataset is compiled by Queensland Treasury, on the basis of aggregate tax records provided by each Australian state or territory government.

### Measures

The following measures were extracted from each problem gambling prevalence study: a) the prevalence of ‘problem gambling’, where problem gambling was defined as Problem Gambling Severity Index (PGSI) ≥ 8 or South Oaks Gambling Screen (SOGS) ≥ 5, b) the prevalence of ‘moderate-risk problem gambling’, where moderate-risk problem gambling was defined as PGSI 3 – 7 or SOGS 3 – 4, c) the jurisdiction, d) the year during which data was collected, e) the administration mode of the survey, i.e. telephone or face-to-face, f) whether the SOGS [25] or the PGSI [26] was used to assess problem gambling, g) the sample size of the survey, and h) any ‘frequency threshold’ used to select which respondents would be administered the problem gambling screen. A frequency threshold is a rule by which the problem gambling screening instrument is only administered to a subset of respondents, selected on the basis of their reported gambling frequency. For example, the screen may only be administered to those who gamble at least weekly, with less frequent gamblers being imputed a problem gambling score of zero. The prevalence of problem gambling and the prevalence of moderate-risk problem gambling were the key outcome variables of interest.

Problem gambling screen and administration mode variables were coded as dummy variables. The gambling frequency threshold variable was collapsed into four categories: weekly gambling, fortnightly gambling, monthly gambling and less than monthly gambling. The year the survey was conducted was subtracted from 2015 to calculate the age of the reported survey.

The measure of EGM gambling losses used was the sum of EGM gambling losses in hotels and clubs and all gambling losses in casinos, both expressed as a percentage of total household disposable income (HDI). This is an aggregate measure, with a single number reported for each state and territory in each year. It is calculated by expressing the total gambling expenditure on EGMs and in casinos for the jurisdiction as a percentage of HDI for the jurisdiction, where HDI is the total income accruing to the household sector less household sector taxes. HDI for each state and territory is reported annually in the *Australian System of National Accounts* [27]. Because gambling losses were reported for fiscal years (spanning 1 July – 30 June) while survey dates were recorded for calendar years, losses for each calendar year were estimated by calculating the mean of losses for the two overlapping fiscal years.

### Statistical analysis

Random effects meta-regression was used to estimate the partial correlation between problem gambling prevalence and EGM and casino losses, after adjusting for methodological variations in prevalence studies and the year of the survey. Meta-regression is an extension of meta-analysis, but has different aims. In general, the goal of a meta-analysis is to pool the varying results of primary studies and thereby arrive at a more accurate and precise estimate of a quantity of interest (e.g. the population prevalence of problem gambling). In contrast, a meta-regression analysis aims to understand what causes variation in the findings of primary studies, using procedures developed for regression analysis [28]. In this context, we might interpret this study as contributing to an ‘epidemiology of problem gambling prevalence studies’ [29].

A random effects model is a statistical extension to fixed effects meta-regression. While a fixed effects analysis relies on the assumption that the quantity of interest (e.g. the population prevalence of problem gambling) is truly consistent across all studies even if it is imperfectly measured, this is rarely the case. For example, the populations under study are unlikely to be identical in all relevant factors, even after covariates are adjusted for. A random effects specification is more conservative because it does not make this strong assumption. When applied, random effects meta-regression usually results in estimates with wider confidence intervals than fixed effects meta-regression [28].

The adopted statistical approach modelled the estimated prevalence of problem gambling and moderate-risk problem gambling in each study as a function of the following predictor variables: EGM gambling losses in the jurisdiction; problem gambling screen; administration mode; frequency threshold; and survey year. Variance inflation factors for predictor variables were all less than 4.0. A binomial model specification with a logistic link function was used. Following Higgins and Thompson and Borenstein and colleagues [28, 30], the random effects meta-regression model was specified as follows:

$$ {y}_i\sim \mathrm{Binomial}\left({p}_i,{\  ssize}_i\right) $$

$$ logit\left({p}_i\right)=\alpha +{\beta}_k{\bullet X}_{i k}+{\theta}_i $$

$$ {\theta}_i\sim \mathrm{N}\left(0,\frac{1}{\tau^2}\right) $$

$$ \tau \sim \mathrm{Uniform}\left(0,10\right) $$

$$ {w}_i=\frac{y_i\left(\ {ssize}_i\colorbox[rgb]{1,1,0}{$-$}{y}_i\right)}{{\  ssize}_i} $$

$$ {\sigma}^2=\frac{\sum_i{w}_i\ \left( n-1\right)}{{\left(\sum_i{w}_i\right)}^2-\sum_i{w}_i^2} $$

$$ {I}^2=\frac{\tau^2}{\tau^2+{\sigma}^2} $$

where: *y*_*i*_ is the number of problem gamblers identified in study *i*; *ssize*_*i*_ is the sample size of study *i*; *α* is a constant intercept; *X*_*ik*_is a matrix of *k* predictor variables and *β*_*k*_ is a commensurate vector of estimated regression coefficients including EGM gambling losses, study year and methodological variations; *θ*_*i*_ is a normally distributed random effect with a standard deviation between studies of *τ* (or equivalently a precision of $\frac{1}{\tau^2}$); *n* is the number of studies under analysis; and *I* ^2^ is the proportion of residual variation in the estimates of problem gambling prevalence that is due to heterogeneity between studies (rather than sampling variation). The uniform prior distribution ranging from 0.0 to 10.0 for *τ* was specified on the basis of the simulations carried out by Lambert and colleagues [31]. All models were implemented using ‘gold standard’ [30] fully Bayesian estimation with *R* and *JAGS* [32, 33]. JAGS code listings for all model specifications are available in Additional file 1 (see listings 1 – 6).

The estimates of *β* coefficients were modelled in three different ways in order to test the robustness of results to the provision of prior information about their values. In the first model, all *β* coefficients were estimated from the prevalence study data set in the usual manner of regression analysis, using ‘weakly informative’ priors distributions. This method has the advantage of minimising residual heterogeneity, but risks identifying spurious correlations [34]. In the second model, coefficients – except for the intercept, the EGM loss coefficient and the year of survey coefficient – were ‘fixed’ on the basis of estimates derived from the small literature concerned with their estimation. This is equivalent to the recent practice that has been applied to compare prevalence estimates between studies e.g. [8, 13], where prevalence estimates are normalised by multiplying them by fixed adjustment factors. Fixing coefficients is advantageous as it forces control variables to be set at plausible values and reduces the effective degrees of freedom of the models. However, it admits no variance in coefficient estimates. Consequently, a third method which constitutes a compromise between the first two was also adopted, assigning ‘informative’ prior distributions to control variable parameter coefficients, regularising them within plausible ranges while still admitting uncertainty in their estimated values.

Prior distributions for *β* coefficients in all three models are listed in Table 1. Weakly informative priors were placed on the *β* coefficients for HDI loss and year of survey in all models. The standard deviations for the informative priors were inflated by a factor of four compared to those derived from meta-analysis, on the basis that the small number of studies synthesised in the meta-analyses were likely to lead to an over-estimation of the precision of these distributions. The unpublished meta-analyses that formed the basis of the informative prior distributions are attached as Additional file 2. Each meta-regression model was estimated twice, first with the prevalence of problem gambling as the outcome variable, and second with the prevalence of moderate-risk problem gambling as the outcome variable.

**Table 1:** Prior distributions placed on meta-regression parameter coefficients

	Models of problem gambling			Models of moderate-risk problem gambling		
	Weakly informative	Informative	Fixed	Weakly informative	Informative	Fixed
Intercept	*N*(0.00, 10^12^)	*N*(0.00, 10^12^)	*N*(0.00, 10^12^)	*N*(0.00, 10^12^)	*N*(0.00, 10^12^)	*N*(0.00, 10^12^)
% HDI lost on EGMs and at casinos	*N*(0.00, 10^12^)	*N*(0.00, 10^12^)	*N*(0.00, 10^12^)	*N*(0.00, 10^12^)	*N*(0.00, 10^12^)	*N*(0.00, 10^12^)
Years before 2015	*N*(0.00, 10^12^)	*N*(0.00, 10^12^)	*N*(0.00, 10^12^)	*N*(0.00, 10^12^)	*N*(0.00, 10^12^)	*N*(0.00, 10^12^)
Administered face-to-face	*N*(0.00, 10^12^)	*N*(0.08, 1.60)	*N*(0.08, 10^−48^)	*N*(0.00, 10^12^)	*N*(0.73, 0.71)	*N*(0.73, 10^−48^)
Used SOGS	*N*(0.00, 10^12^)	*N*(0.48, 0.40)	*N*(0.48, 10^−48^)	*N*(0.00, 10^12^)	*N*(−0.53, 0.40)	*N*(−0.53, 10^−48^)
Monthly frequency threshold	*N*(0.00, 10^12^)	*N*(−0.03, 0.46)	*N*(−0.03, 10^−48^)	*N*(0.00, 10^12^)	*N*(−0.14, 0.23)	*N*(−0.14, 10^−48^)
Fortnightly frequency threshold	*N*(0.00, 10^12^)	*N*(−0.10, 0.40)	*N*(−0.10, 10^−48^)	*N*(0.00, 10^12^)	*N*(−0.38, 0.22)	*N*(−0.38, 10^−48^)
Weekly frequency threshold	*N*(0.00, 10^12^)	*N*(−0.40, 0.37)	*N*(−0.40, 10^−48^)	*N*(0.00, 10^12^)	*N*(−0.56, 0.24)	*N*(−0.56, 10^−48^)

These models address the two research objectives of this study. First, the player loss *β* coefficient can be interpreted as an indicator of the magnitude and direction of any association between aggregate EGM gambling losses and the prevalence of problem gambling. Second, the *I* ^2^ estimate describes the degree of heterogeneity that remains among problem gambling prevalence estimates *after* accounting for both sampling variability within individual prevalence studies and the predictor variables described above. In other words, *I* ^2^ measures the inconstancy in prevalence estimates between studies rather than real variation in the prevalence of problem gambling. *I* ^2^ has a range of 0.0 to 1.0, with Higgins and colleagues suggesting that the values of 0.25, 0.5, and 0.75 indicate low, moderate and high degrees of heterogeneity respectively [35].

## Results

### Selection

A total of 45 problem gambling prevalence estimates were identified, including eight state-level estimates derived the Productivity Commission’s 1999 national survey [see Additional file 3, Fig. S1 and Table S1]. Once ineligible studies were excluded, 41 studies were identified that estimated the prevalence of problem gambling, 40 of which also estimated the prevalence of moderate-risk problem gambling. All extracted data for each study are listed in Table 2.

**Table 2:** Problem gambling prevalence studies which met the eligibility criteria

State or territory	Year	Sample size	Prevalence of problem gambling (% of adults)	Prevalence of moderate-risk problem gambling (% of adults)	Administration mode	Screen	Gambling frequency threshold	Losses on EGMs and at casinos (% of HDI)
ACT	1999	708	2.06	2.54	Telephone	SOGS	Weekly	1.768
ACT	2001	5445	1.91	1.21	Telephone	SOGS	Weekly	1.587
ACT	2009	5500	0.50	1.50	Telephone	PGSI	Monthly	0.781
ACT	2014-15	7068	0.40	1.10	Telephone	PGSI	Six-monthly or less often	0.594
NSW	1995	1390	2.20	3.08	Doorknock	SOGS	Weekly	1.952
NSW	1997	1209	3.00	4.14	Doorknock	SOGS	Weekly	2.247
NSW	1999	2632	2.55	2.57	Telephone	SOGS	Weekly	2.726
NSW	2006	5029	0.80	1.60	Telephone	PGSI	Weekly	2.570
NSW	2008-09	9408	0.40	1.30	Telephone	PGSI	Six-monthly or less often	2.082
NSW	2011	10,000	0.80	2.90	Telephone	PGSI	Six-monthly or less often	1.944
NT	1999	607	1.89	0.42	Telephone	SOGS	Weekly	2.009
NT	2005	5246	0.64	1.57	Telephone	PGSI	Weekly	2.129
QLD	1999	1518	1.88	4.13	Telephone	SOGS	Weekly	1.888
QLD	2001	13,082	0.83	2.70	Telephone	PGSI	Six-monthly or less often	1.948
QLD	2003-04	30,373	0.55	1.97	Telephone	PGSI	Six-monthly or less often	2.105
QLD	2006-07	30,188	0.47	1.80	Telephone	PGSI	Six-monthly or less often	1.608
QLD	2008-09	14,962	0.37	1.60	Telephone	PGSI	Six-monthly or less often	1.460
QLD	2011-12	15,088	0.48	1.90	Telephone	PGSI	Six-monthly or less often	1.346
SA	1996	1206	1.24		Telephone	SOGS	Weekly	1.520
SA	1999	1013	2.45	0.57	Telephone	SOGS	Weekly	1.824
SA	2001	6045	1.88	1.36	Telephone	SOGS	Monthly	1.886
SA	2005	17,140	0.40	1.20	Telephone	PGSI	Fortnightly	1.992
SA	2012	9508	0.60	2.50	Telephone	PGSI	Six-monthly or less often	1.370
TAS	1994	1220	0.82	1.97	Doorknock	SOGS	Weekly	0.811
TAS	1996	1211	2.89	5.70	Telephone	SOGS	Six-monthly or less often	0.934
TAS	1999	810	0.44	1.73	Telephone	SOGS	Weekly	1.609
TAS	2000	1223	0.90	1.55	Telephone	SOGS	Six-monthly or less often	1.743
TAS	2005	6048	0.73	1.02	Telephone	PGSI	Weekly	1.715
TAS	2007	4051	0.54	0.86	Telephone	PGSI	Weekly	1.438
TAS	2011	4303	0.70	1.80	Telephone	PGSI	Six-monthly or less often	1.189
TAS	2013	5000	0.50	1.80	Telephone	PGSI	Six-monthly or less often	1.052
VIC	1997	2000	1.00	1.30	Telephone	SOGS	Six-monthly or less often	2.489
VIC	1998	1737	1.50	1.10	Telephone	SOGS	Six-monthly or less often	2.707
VIC	1999a	1760	0.80	1.30	Telephone	SOGS	Six-monthly or less often	2.843
VIC	1999b	2227	2.14	2.65	Telephone	SOGS	Weekly	2.843
VIC	2003	8479	0.97	0.91	Telephone	PGSI	Weekly	2.598
VIC	2007	2012	1.40	2.80	Telephone	PGSI	Six-monthly or less often	2.175
VIC	2008	15,000	0.70	2.36	Telephone	PGSI	Six-monthly or less often	2.083
VIC	2014	13,554	0.81	2.79	Telephone	PGSI	Six-monthly or less often	1.748
WA	1994	1253	0.56	0.48	Doorknock	SOGS	Weekly	1.285
WA	1999	1114	0.70	4.15	Telephone	SOGS	Weekly	0.758

Notes: *SOGS* South Oaks Gambling Screen, *PGSI* Problem Gambling Severity Index. Full bibliographic details for each study can be found in the Additional file 3 as Table S1

Just over half of studies identified used the PGSI (*n* = 21), with the remainder using SOGS. The vast majority were administered by telephone (*n* = 37). The most commonly used gambling frequency thresholds for the administration of a problem gambling screen were a weekly threshold (*n* = 19) and the combined category of an annual threshold, a six-monthly threshold or no threshold at all (*n* = 19). Only two studies used a monthly threshold, while a single study used a fortnightly threshold. The median sample size was 4303 (*Inter-quartile range [IQR]* = 1253 – 9408), with a total of 267,367 adults responding to the 41 surveys. The median survey year was 2001 (*IQR* = 1999 – 2008).

### Outcome data and main results

The average non-regularised prevalence of problem gambling across all studies was 0.9% of adults [95% Credible Interval (Cr.I.) 0.8%, 1.1%], with the average non-regularised prevalence of moderate-risk problem gambling estimated to be 1.8% of adults (95% Cr.I. 1.5%, 2.1%) (see Additional file 3, Fig. S2 and S3). There was an extraordinarily large degree of heterogeneity among these studies, with *I* ^2^ for problem gambling and moderate-risk problem gambling estimated at 0.95 (95% Cr.I. 0.94, 0.95) and 0.94 (95% Cr.I. 0.93, 0.96) respectively.

**Fig. 1:**
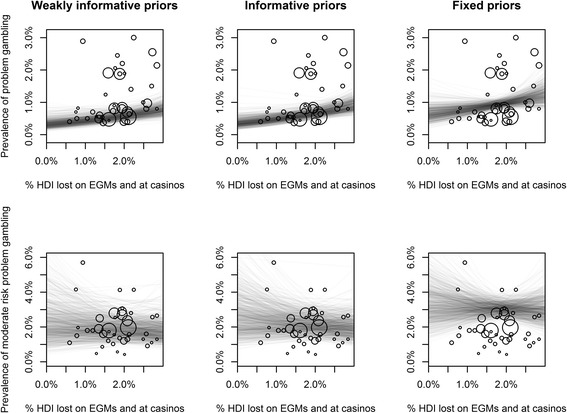
Posterior estimates of the association between prevalence and money lost gambling on EGMs and at casinos. Each point represents a prevalence study, with *circles sized* proportionately with their weight in the meta-regression analyses. Each *line* indicates a single draw from the estimated model. *HDI* Household Disposable Income, *EGMs* Electronic Gaming Machines

An association between the prevalence of problem gambling and EGM and casino gambling losses was apparent in the meta-regression model with weakly informative priors. Parameter coefficients, expressed as ‘prevalence ratios’ are displayed in Table 3. Prevalence ratios should be interpreted analogously to incidence rate ratios, and can be multiplied with the intercept to predict the value of the outcome variable for a given set of predictor variable values. Every increase of 1 % of household disposable income lost on EGMs and at casinos was associated with problem gambling prevalence estimates that were 1.35 (95% Cr.I. 1.04, 1.74) times higher. Placing informative priors on other meta-regression coefficients decreased the parameter estimate slightly to 1.33 (95% Cr.I. 1.04 1.71). Fixing coefficients related to methodological variations in prevalence studies slightly reduced the estimated association between prevalence and EGM and casino gambling losses and decreased the precision of the estimate, bringing the ‘no association’ prevalence ratio of 1.0 to within the 95% credible interval (1.29, 95% Cr.I. 0.98, 1.72). Posterior estimates of the associations between prevalence and losses are visualised in Fig. 1.

**Table 3:** Meta-regression analyses of the prevalence of problem gambling and moderate-risk problem gambling. Parameter estimates have been exponentiated and should be interpreted as prevalence ratios, analogous to odds ratios

Estimates of *α* and *β* coefficients and descriptive statistics	Problem gambling			Moderate-risk problem gambling		
	Weakly informative	Informative	Fixed	Weakly informative	Informative	Fixed
Intercept	**0.004 [0.002, 0.006]**	**0.004 [0.002, 0.006]**	**0.003 [0.002, 0.006]**	**0.021 [0.011, 0.042]**	**0.019 [0.010, 0.036]**	**0.016 [0.008, 0.032]**
% HDI lost on EGMs and at casinos	**1.35 [1.04, 1.74]**	**1.33 [1.04, 1.71]**	1.29 [0.98, 1.72]	0.94 [0.66, 1.34]	0.95 [0.68, 1.32]	0.97 [0.69, 1.37]
Years before 2015	0.99 [0.94, 1.04]	1.00 [0.96, 1.05]	**1.04 [1.02, 1.07]**	1.00 [0.93, 1.08]	1.02 [0.96, 1.08]	**1.05 [1.02, 1.09]**
Administered face-to-face	1.02 [0.59, 1.78]	1.02 [0.60, 1.74]	1.08^a^	1.17 [0.55, 2.53]	1.33 [0.71, 2.55]	2.07^a^
Used SOGS	**2.37 [1.37, 4.00]**	**2.18 [1.42, 3.34]**	1.62^a^	1.20 [0.57, 2.52]	0.99 [0.55, 1.76]	0.59^a^
Monthly frequency threshold	1.26 [0.68, 2.34]	1.15 [0.68, 1.84]	0.97^a^	0.67 [0.28, 1.58]	0.81 [0.54, 1.20]	0.87^a^
Fortnightly frequency threshold	0.65 [0.28, 1.50]	0.74 [0.43, 1.31]	0.91^a^	0.65 [0.21, 2.15]	0.67 [0.45, 1.01]	0.68^a^
Weekly frequency threshold	1.21 [0.86, 1.66]	1.10 [0.82, 1.47]	0.67^a^	0.77 [0.49, 1.20]	**0.68 [0.50, 0.94]**	0.57^a^
*τ*	0.37 [0.26, 0.51]	0.37 [0.25, 0.50]	0.46 [0.35, 0.60]	0.55 [0.40, 0.73]	0.53 [0.39, 0.70]	0.59 [0.44, 0.75]
*R* ^2^	0.65 [0.29, 0.89]	0.66 [0.31, 0.88]	0.47 [0.00, 0.71]	0.00 [0.00, 0.41]	0.00 [0.00, 0.44]	0.00 [0.00, 0.30]
*I* ^2^	0.87 [0.78, 0.93]	0.86 [0.78, 0.93]	0.91 [0.86, 0.95]	0.97 [0.96, 0.99]	0.97 [0.95, 0.99]	0.98 [0.96, 0.99]

Notes: 95% credible intervals are indicated in square brackets. Estimates where the 95% credible interval does not include 1.0 are indicated in boldface, except where coefficients are fixed. ^a^ indicates a coefficient that is fixed a priori rather than being estimated from the data. *n* = 41 for problem gambling and *n* = 40 for moderate-risk problem gambling. Problem gambling is defined as a PGSI score of 8 or more or a SOGS score of 5 or more. Moderate-risk problem gambling is defined as a PGSI score of 3 – 7 or a SOGS score of 3 – 4. *HDI* household disposable income, *EGM* Electronic Gaming Machine, *SOGS* South Oaks Gambling Screen, *PGSI* Problem Gambling Severity Index

Few clear associations were found between moderate-risk problem gambling and any predictor variables using the models with weakly informative priors. With the use of informative priors, only the estimated association between moderate-risk problem gambling prevalence and a weekly gambling frequency threshold became reasonably precise (0.68, 95% Cr.I. 0.50, 0.94).

The number of years the study was conducted before 2015 was positively associated with both problem gambling prevalence and moderate-risk problem gambling prevalence, but only when meta-regression coefficients were fixed at the values arrived at from meta-analyses of previous within-study estimates. Models with fixed priors found that a 1 year increase in the age of the study was associated with prevalence estimates that were 1.04 (95% Cr.I. 1.02, 1.07) times greater for problem gambling and 1.05 (95% Cr.I. 1.02, 1.09) times greater for moderate-risk problem gambling.

A great deal of residual heterogeneity was evident among all models. While the models of problem gambling prevalence explained up to 66% of the variation among estimates, *I* ^2^ for these data fell in the range between 0.78 and 0.95. This means that even after adjusting for covariates, a great deal of variation remained among problem gambling prevalence estimates, with unexplained heterogeneity between studies dominating random sampling error. This unsatisfactory situation was more extreme for estimates of moderate-risk problem gambling. No model explained even 1% of the variation among moderate-risk problem gambling prevalence estimates. The lower bound of estimates of *I* ^2^ for models of moderate-risk problem gambling was 0.95.

## Discussion

The study had three key findings. First, problem gambling prevalence was associated with EGM and casino gambling losses in models with informative and weakly informative priors. An increase of 1% of household disposable income spend on EGMs and casino gambling associated with prevalence estimates that were approximately 1.3 times greater. In models where control parameter coefficients were fixed at values derived from meta-analyses, the point estimates of the gambling loss coefficient were similar but the 95% credible intervals widened to include a prevalence ratio of 1.0. In short, these results support the total consumption theory of gambling, but should be interpreted cautiously, given the degree of uncertainty evident in estimates.

The relatively wide uncertainty interval surrounding this finding is unsurprising given the relatively modest number of studies (*n* = 41) and their high degree of heterogeneity with respect to problem gambling prevalence (*I* ^2^ > 0.85 in all cases). Consequently, this study provides only a moderate degree of confidence that EGM gambling losses and problem gambling prevalence estimates are correlated. The best-fitting model, which used informative priors, suggested that an increase in EGM gambling losses of 1% of HDI is associated with a population-level increase in problem gambling prevalence of around 1.33 times (95% Cr.I. 1.04 – 1.71). The width of this uncertainty band is likely to be a consequence of measurement error overwhelming true variation in problem gambling prevalence. This high level of statistical noise in measurement is likely to derive from methodological variations that were unaccounted for in this study. For example, we did not adjust for way the survey was described to potential respondents, a factor that can impact non-response bias [36]. Future studies using more consistently-collected data could seek to measure the relationship between EGM losses and problem gambling prevalence with more precision. These results support the need to phase out state-based prevalence studies and transition to national problem gambling prevalence studies that are adequately powered to investigate individual jurisdictions and that remain methodologically stable over time [16].

The moderate degree of uncertainty remaining around the association between EGM losses and harm should be interpreted in the context of parallel findings at other spatial scales. EGM gambling losses are correlated with risk of developing gambling problems for individuals [23] and for populations aggregated by county, school or gambling venue [4, 5]. The present study adds to the weight of evidence that an increase in population losses on EGMs is associated with an increase in the prevalence of problem gambling.

The second key finding of the study was that a high degree of heterogeneity exists in problem gambling prevalence estimates. Only a moderate degree of variation among prevalence estimates was explained by EGM gambling losses, methodological variations, or year of study (*R* ^2^ ≤ 0.66). When coefficient values for methodological variations were fixed based on prior research, variance explained fell substantially (*R* ^2^ = 0.47). Very little of the residual variation between problem gambling prevalence estimates was due to sampling error, with a very high degree of unexplained heterogeneity (*I* ^2^ ≥ 0.86). Put differently, after adjusting for methodological variations, EGM losses and year of survey, no more than 14% of the residual differences between problem gambling prevalence estimates results from sampling error. These results raise questions about the validity of comparing problem gambling prevalence estimates, even after adjusting for methodological variations between studies.

The third key finding was the absence of any apparent pattern among moderate-risk problem gambling prevalence estimates. Contrary to expectations premised on total consumption theory, no link was evident between moderate-risk problem gambling prevalence estimates and EGM and casino gambling losses. Furthermore, no model explained any meaningful amount of the variation in moderate-risk problem gambling prevalence estimates, with the point estimate of *R* ^2^ falling below 0.01 in all cases. In other words, none of the models explained even 1% of the variation in estimates of moderate-risk problem gambling.

The lack of an apparent relationship between EGM gambling losses and moderate-risk problem gambling prevalence has two potential interpretations. It could be that there is no real relationship between moderate-risk problem gambling and EGM spending, in contradiction to total consumption theory. Alternatively, it is also plausible that the PGSI and SOGS are mismeasuring the population at moderate risk of problem gambling. Given that problem gambling screens have been developed and validated to identify problem or pathological gamblers [26], it may be that problem gambling screens are not fit for the purpose of identifying moderate-risk problem gamblers.

Several pieces of evidence offer tentative support for the mismeasurement conjecture. First, the only published validation study of the moderate-risk classification of the PGSI found that it lacked discriminant validity [37]. In particular, this study found no practical differences between those scoring 1-2 on the PGSI and those scoring 3-7. Indeed, as McCready and Adlaf [38] note, the PGSI does not include any items designed to discriminate among gamblers with less severe problems. As the authors of the PGSI acknowledge in their original study, the screen’s division between low- and moderate-risk gambling categories is only tentatively supported by the survey data from which it was derived [26]. In addition, the explanatory power of the variables included in the meta-regression analysis for predicting moderate-risk problem gambling prevalence estimates was exceptionally poor, with *R* ^2^ < 0.01 in all three models (Table 3). Similarly, residual heterogeneity was very high, with *I* ^2^ ≥ 0.97 across all models. This implies that either moderate-risk problem gambling prevalence estimates are not impacted by methodological variations, or that such impacts are very small when compared to other unaccounted for factors. Finally, the meta-analysis of problem gambling screen effects presented in Additional file 2 finds a much greater degree of heterogeneity is present in estimates of screen impacts on moderate-risk problem gambling prevalence estimates (*I* ^2^ = 0.91) than problem gambling prevalence estimates (*I* ^2^ = 0.69). In other words, the ‘within study’ estimates of the impact of methodological variations on prevalence estimates vary a great deal *between* studies of moderate-risk problem gambling.

Taken together, this suggests that measures of moderate-risk problem gambling are extremely imprecise, to the point of possibly being the equivalent of statistical white noise. The apparent inability of current screening instruments to reliably identify this population is particularly problematic given that recent evidence suggests that this population experience the greatest mass of gambling-related harms when measured in terms of disability-adjusted life years [39].

These results are subject to several limitations. First, a great deal of variation existed among prevalence estimates after adjusting for five predictor variables. Thus, estimates of any association between prevalence and EGM losses are necessarily imprecise. Second, the 41 prevalence estimates analysed in this study may be insufficient for the assessment of population trends, especially among the moderate-risk problem gambler population. Third, other methodological variations that this study was unable to adjust for may impact on problem gambling prevalence estimates. Finally, it is possible that some individuals may have been sampled in surveys in multiple years, especially in smaller jurisdictions. This is unlikely to have a substantial effect on the results.

## Conclusions

This study has three key implications. First, the finding of an association between EGM and casino gambling losses and problem gambling prevalence is consistent with total consumption theory. Therefore, interventions by jurisdictional governments that reduce total EGM gambling losses among the whole population are likely to effectively reduce the prevalence of problem gambling. This result was evident despite the imprecision and heterogeneity of these estimates. Nevertheless, replication using a large cross-jurisdictional surveys that are consistent over time are required to confirm this association with a greater degree of confidence.

Second, this study demonstrates that using single-jurisdiction prevalence studies for making comparisons between jurisdictions or within the same jurisdiction over time is ineffectual [17]. Even after deploying sophisticated statistical adjustments, the high degree of residual heterogeneity evident in this study suggests that the validity of comparing problem gambling prevalence estimates may be poor. The situation is far worse for moderate-risk problem gambling. It appears that although 267,367 Australian adults have responded to 41 surveys, we still are unable to confidently compare problem gambling prevalence either between jurisdictions or over time, a situation that is likely to be replicated internationally. It will be necessary to undertake adequately-powered, multi-jurisdictional prevalence studies – with survey instruments and data collection protocols that remain consistent over time – if the scientific value of problem gambling prevalence studies is to be increased in future.

Third, this study suggests that the suitability of PGSI and SOGS for estimating the population prevalence of moderate-risk problem gambling needs urgent investigation. The validity and reliability of the moderate-risk problem gambling classification is unclear. No patterns were evident among the estimates of the population prevalence of this group between studies, symptomatic of either extreme measurement errors or poor construct validity. The interpretation of moderate-risk problem gambling prevalence estimates should only be undertaken with extreme caution until a greater degree of conceptual and statistical clarity is brought to the identification of this population.

## Additional files

Additional file 1: JAGS models. JAGS code listings for all six of the models presented in this paper.c. (PDF 513 kb)

Additional file 2: Meta-analyses of previous studies of methodological variations. An unpublished manuscript which seeks to estimate the average impact of the following methodological variations of prevalence estimates: (a) choice of problem gambling screen, (b) survey administration mode, or (c) choice of frequency threshold. (PDF 902 kb)

Additional file 3: Supplementary figures and tables. Study inclusion flow diagram; problem gambling prevalence forest plot; moderate risk problem gambling prevalence forest plot; and full bibliographic details for each study. (PDF 1173 kb)

## Abbreviations

Cr. I.: Credible Interval
EGM: Electronic gaming machine
HDI: Household disposable income
IQR: Inter-quartile range
PGSI: Problem gambling severity index
SOGS: South oaks gambling screen
