# Supplementary material for: A meta-regression analysis of 41 Australian problem gambling prevalence estimates and their relationship to total spending on electronic gaming machines
Source: BMC Public Health. 2017 May 23;17:495. doi: 10.1186/s12889-017-4413-6 (PMC5442595; doi:10.1186/s12889-017-4413-6)
Supplement: Supplementary file 1 — JAGS models. JAGS code listings for all six of the models presented in this paper.c. (PDF 513 kb) [file 12889_2017_4413_MOESM1_ESM.pdf]

Additional file 1: JAGS models

A meta-regression analysis of 41 Australian problem gambling prevalence estimates and their relationship to total spending on electronic gaming machines

Authors: Francis Markham [*], Martin Young, Bruce Doran and Mark Sugden

Listing 1: Random effects meta-analysis of the prevalence of problem gambling, without moderators

```
model
      {
         for(i in 1:N) {
            y[i] ~ dbin(p[i], s_size[i])
            logit(p[i]) <- intercept + theta[i]

            # Calculate the weights to help with estimating Q
        w[i] <- 1 / (1/y[i] + 1/(s_size[i] - y[i]))

            theta[i] ~ dnorm(0, prec)
            resid[i] <- y[i] - fitted_est[i]
            fitted_est[i] <- p[i] * s_size[i]
      }

      # Calculate Q from Borenstein et al, p. 114, eq. 16.5
      N_params <- 1

      # Calculate Q from eq 16.1 on p. 109, Borenstein et al.
      Q <- sum(w * ((logit(p) - intercept)^2))

      # Calculate I2
      # As defined on p. 1546, eq. 10 in http://doi.org/10.1002/sim.1186
      H.sq <- Q / (N - N_params)

      # As defined on Borenstein et al., p. 117, eq. 16.9
```

```
      I.sq <- min((Q - (N - N_params)) / max(Q, 10^-12), 1.0) * 100.0

      intercept ~ dnorm(0, 10^-6)

      tau~  dunif(0,10)  # Suggested by http://dx.doi.org/10.1002/sim.2112
      tau.sq <- tau*tau
      prec <- 1/(tau.sq)
      }


# Convenience settings for runjags in R:
#monitor# intercept, tau, tau.sq, Q, I.sq, H.sq
#modules# glm on
#response# y
#residual# resid
#fitted# fitted_est


######################################################################################################
######################################################################################################
#### Initial values
######################################################################################################
######################################################################################################

inits{
"intercept" <- -1.5
}

inits{
"intercept" <- -0.5
}

inits{
"intercept" <- 0.5
}

inits{
"intercept" <- 1.5
}
```

Listing 2: Random effects meta-analysis of the prevalence of problem gambling, with moderators and informative priors

```
model
      {

         for(i in 1:N) {
            y[i] ~ dbin(p[i], s_size[i])
            logit(p[i]) <- intercept +
                  B_doorknock * doorknock[i] +
                  B_SOGS * SOGS[i] +
                  B_freq_thresh_m * freq_thresh_m[i] +
                  B_freq_thresh_f * freq_thresh_f[i] +
                  B_freq_thresh_w * freq_thresh_w[i] +
                  B_Years * Years_before_2016[i] +
                  B_Exp_EGM_pc_hdi * Exp_TG_EGM_pc_hdi[i] +
                  theta[i]

            theta[i] ~ dnorm(0, prec)

            # Inverse variance weights for I2
            w[i] <- 1 / (1/y[i] + 1/(s_size[i] - y[i]))

            # Repeat with no covariates for R2 calc
            y_full[i] ~ dbin(p_full[i], s_size[i])
            logit(p_full[i]) <- intercept_full + theta_full[i]
            theta_full[i] ~ dnorm(0, prec_full)

            # calculate residuals and fitted values to bring back into R
            resid[i] <- y[i] - fitted_est[i]
            fitted_est[i] <- p[i] * s_size[i]
      }

      # Priors
      FLATTEN_FACTOR <- 4

      intercept ~ dnorm(0, 10^-6)
      B_doorknock ~ dnorm(0.0761,(1/(0.3998*FLATTEN_FACTOR))^2)
      B_SOGS ~ dnorm(0.4797, (1/(0.1000*FLATTEN_FACTOR))^2)
```

```
    B_freq_thresh_m ~ dnorm(-0.0332, (1/(0.1150*FLATTEN_FACTOR))^2)
    B_freq_thresh_f ~ dnorm( -0.0958, (1/(0.1002*FLATTEN_FACTOR))^2)
    B_freq_thresh_w ~ dnorm(-0.3965, (1/(0.0915*FLATTEN_FACTOR))^2)
    B_Years ~ dnorm(0, 10^-6)
    B_Exp_EGM_pc_hdi ~ dnorm(0, 10^-6)

    # Heterogeneity statistics
    N_params <- 8

    # Calculate tau and tau2
    prec <- 1/(tau.sq)
    tau.sq <- tau^2
    tau ~ dunif(0,10)  # Suggested by http://dx.doi.org/10.1002/sim.2112

    # Calculate I2
    # As defined on p. 1546, eq. 9 and 10 http://doi.org/10.1002/sim.1186
    H.sq <- (tau.sq + sigma.sq) / sigma.sq
    sigma.sq <- (sum(w) * (N - 1)) / (sum(w)^2 - sum(w^2))
    I.sq <- tau.sq / (tau.sq + sigma.sq)

    # H.sq.v2 <- (((sum(w)  - (sum(w^2) / sum(w))) * tau.sq) / (N - 1)) + 1 # eq. 11 and 10 are equivalent
    # I.sq.v2 <- (H.sq.v2 - 1) / H.sq.v2

    # R2 calc
    intercept_full ~ dnorm(0, 10^-6)
    tau_full ~ dunif(0,10)
    tau.sq_full <- tau_full^2
    prec_full <- 1/(tau.sq_full)
    R.sq <- (1 - max(min(tau.sq / tau.sq_full, 1.0), 0.0)) * 100.0
    }


# Convenience settings for runjags in R:
#monitor# intercept, B_doorknock, B_SOGS, B_Years, B_Exp_EGM_pc_hdi, B_freq_thresh_m, B_freq_thresh_f, B_freq_thresh_w,
tau, tau.sq, tau_full, tau.sq_full, I.sq, H.sq, R.sq
#modules# glm on
#response# y
#residual# resid
#fitted# fitted_est
```

```
######################################################################################################
######################################################################################################
#### Initial values
######################################################################################################
######################################################################################################

inits{
"intercept" <- -1.5
"B_doorknock" <- -1.5
"B_SOGS"  <- 1.5
"B_freq_thresh_m"  <- 0
"B_freq_thresh_f"  <- 0.5
"B_freq_thresh_w"  <- 0
"B_Years"  <- -0.5
"B_Exp_EGM_pc_hdi" <- -0.5
}

inits{
"intercept" <- -0.5
"B_doorknock" <- -0.5
"B_SOGS"  <- -1.5
"B_freq_thresh_m"  <- 0
"B_freq_thresh_f"  <- 0.5
"B_freq_thresh_w"  <- 0
"B_Years"  <- 1.5
"B_Exp_EGM_pc_hdi" <- 1.5

}

inits{
"intercept" <- 0.5
"B_doorknock" <- -0.5
"B_SOGS"  <- 1.5
"B_freq_thresh_m"  <- 0
"B_freq_thresh_f"  <- 0.5
"B_freq_thresh_w"  <- 0
"B_Years"  <- 1.5
```

```
"B_Exp_EGM_pc_hdi" <- -1.5
}

inits{
"intercept" <- 1.5
"B_doorknock" <- -1.5
"B_SOGS"  <-  -0.5
"B_freq_thresh_m"  <- 0
"B_freq_thresh_f"  <- 1.5
"B_freq_thresh_w"  <- 0
"B_Years"  <- -0.5
"B_Exp_EGM_pc_hdi" <- 0.5
}
```

Listing 3: Random effects meta-analysis of the prevalence of problem gambling, with moderators and fixed priors

```
model
      {
         for(i in 1:N) {
            y[i] ~ dbin(p[i], s_size[i])
            logit(p[i]) <- intercept +
                  B_doorknock * doorknock[i] +
                  B_SOGS * SOGS[i] +
                  B_freq_thresh_m * freq_thresh_m[i] +
                  B_freq_thresh_f * freq_thresh_f[i] +
                  B_freq_thresh_w * freq_thresh_w[i] +
                  B_Years * Years_before_2016[i] +
                  B_Exp_EGM_pc_hdi * Exp_TG_EGM_pc_hdi[i] +
                  theta[i]

            theta[i] ~ dnorm(0, prec)

            # Inverse variance weights for I2
            w[i] <- 1 / (1/y[i] + 1/(s_size[i] - y[i]))

            # Repeat with no covariates for R2 calc
            y_full[i] ~ dbin(p_full[i], s_size[i])
            logit(p_full[i]) <- intercept_full + theta_full[i]
            theta_full[i] ~ dnorm(0, prec_full)

            # calculate residuals and fitted values to bring back into R
            resid[i] <- y[i] - fitted_est[i]
            fitted_est[i] <- p[i] * s_size[i]
      }

      # Priors
      intercept ~ dnorm(0, 10^-6)
      B_doorknock ~ dnorm(0.0761, 10^24)
      B_SOGS ~ dnorm(0.4797, 10^24)
      B_freq_thresh_m ~ dnorm(-0.0332, 10^24)
      B_freq_thresh_f ~ dnorm( -0.0958, 10^24)
      B_freq_thresh_w ~ dnorm(-0.3965, 10^24)
```

```
    B_Years ~ dnorm(0, 10^-6)
    B_Exp_EGM_pc_hdi ~ dnorm(0, 10^-6)


    # Heterogeneity statistics
    N_params <- 8

    # Calculate tau and tau2
    prec <- 1/(tau.sq)
    tau.sq <- tau^2
    tau ~ dunif(0,10)  # Suggested by http://dx.doi.org/10.1002/sim.2112

    # Calculate I2
    # As defined on p. 1546, eq. 9 and 10 http://doi.org/10.1002/sim.1186
    H.sq <- (tau.sq + sigma.sq) / sigma.sq
    sigma.sq <- (sum(w) * (N - 1)) / (sum(w)^2 - sum(w^2))
    I.sq <- tau.sq / (tau.sq + sigma.sq)

    # R2 calc
    intercept_full ~ dnorm(0, 10^-6)
    tau_full ~ dunif(0,10)
    tau.sq_full <- tau_full^2
    prec_full <- 1/(tau.sq_full)
    R.sq <- (1 - max(min(tau.sq / tau.sq_full, 1.0), 0.0)) * 100.0
    }


# Convenience settings for runjags in R:
#monitor# intercept, B_doorknock, B_SOGS, B_Years, B_Exp_EGM_pc_hdi, B_freq_thresh_m, B_freq_thresh_f, B_freq_thresh_w,
tau, tau.sq, tau_full, tau.sq_full, I.sq, H.sq, R.sq
#modules# glm on
#response# y
#residual# resid
#fitted# fitted_est


###########################################################################################################
###########################################################################################################
#### Initial values
```

```
#######################################################################################################
#######################################################################################################

inits{
"intercept" <- -1.5
"B_doorknock" <- -1.5
"B_SOGS"  <- 1.5
"B_freq_thresh_m"  <- 0
"B_freq_thresh_f"  <- 0.5
"B_freq_thresh_w"  <- 0
"B_Years"  <- -0.5
"B_Exp_EGM_pc_hdi" <- -0.5
}

inits{
"intercept" <- -0.5
"B_doorknock" <- -0.5
"B_SOGS"  <- -1.5
"B_freq_thresh_m"  <- 0
"B_freq_thresh_f"  <- 0.5
"B_freq_thresh_w"  <- 0
"B_Years"  <- 1.5
"B_Exp_EGM_pc_hdi" <- 1.5

}

inits{
"intercept" <- 0.5
"B_doorknock" <- -0.5
"B_SOGS"  <- 1.5
"B_freq_thresh_m"  <- 0
"B_freq_thresh_f"  <- 0.5
"B_freq_thresh_w"  <- 0
"B_Years"  <- 1.5
"B_Exp_EGM_pc_hdi" <- -1.5
}

inits{
"intercept" <- 1.5
```

```
"B_doorknock" <- -1.5
"B_SOGS"  <-  -0.5
"B_freq_thresh_m"  <- 0
"B_freq_thresh_f"  <- 1.5
"B_freq_thresh_w"  <- 0
"B_Years"  <- -0.5
"B_Exp_EGM_pc_hdi" <- 0.5
}
```

Listing 4: Random effects meta-analysis of the prevalence of moderate risk problem gambling, without moderators

```
model
      {
         for(i in 1:N) {
            y[i] ~ dbin(p[i], s_size[i])
            logit(p[i]) <- intercept + theta[i]

            # Calculate the weights to help with estimating Q
        w[i] <- 1 / (1/y[i] + 1/(s_size[i] - y[i]))

            theta[i] ~ dnorm(0, prec)
            resid[i] <- y[i] - fitted_est[i]
            fitted_est[i] <- p[i] * s_size[i]
      }

      # Calculate Q from Borenstein et al, p. 114, eq. 16.5
      N_params <- 1
      #Q <- (N - N_params) + C * tau.sq

      # Calculate Q from eq 16.1 on p. 109, Borenstein et al.
      Q <- sum(w * ((logit(p) - intercept)^2))

      # Calculate I2
      # As defined on p. 1546, eq. 9 and 10 http://doi.org/10.1002/sim.1186
      H.sq <- Q / (N - N_params)
      # I.sq.b <- min((H.sq - 1.0) / max(H.sq, 10^-12), 1.0) * 100

      # As defined on Borenstein et al., p. 117, eq. 16.9
      I.sq <- min((Q - (N - N_params)) / max(Q, 10^-12), 1.0) * 100.0

      intercept ~ dnorm(0, 10^-6)

      tau~  dunif(0,10)  # Suggested by http://dx.doi.org/10.1002/sim.2112
      tau.sq <- tau*tau
      prec <- 1/(tau.sq)
      }
```

```
# Convenience settings for runjags in R:
#monitor# intercept, tau, tau.sq, Q, I.sq, H.sq
#modules# glm on
#response# y
#residual# resid
#fitted# fitted_est


######################################################################################################
######################################################################################################
#### Initial values
######################################################################################################
######################################################################################################

inits{
"intercept" <- -1.5
}

inits{
"intercept" <- -0.5
}

inits{
"intercept" <- 0.5
}

inits{
"intercept" <- 1.5
}
```

Listing 5: Random effects meta-analysis of the prevalence of moderate risk problem gambling, with moderators and informative priors

```
model
      {

         for(i in 1:N) {
            y[i] ~ dbin(p[i], s_size[i])
            logit(p[i]) <- intercept +
                  B_doorknock * doorknock[i] +
                  B_SOGS * SOGS[i] +
                  B_freq_thresh_m * freq_thresh_m[i] +
                  B_freq_thresh_f * freq_thresh_f[i] +
                  B_freq_thresh_w * freq_thresh_w[i] +
                  B_Years * Years_before_2016[i] +
                  B_Exp_EGM_pc_hdi * Exp_TG_EGM_pc_hdi[i] +
                  theta[i]

            theta[i] ~ dnorm(0, prec)

            # Inverse variance weights for I2
            w[i] <- 1 / (1/y[i] + 1/(s_size[i] - y[i]))

            # Repeat with no covariates for R2 calc
            y_full[i] ~ dbin(p_full[i], s_size[i])
            logit(p_full[i]) <- intercept_full + theta_full[i]
            theta_full[i] ~ dnorm(0, prec_full)

            # calculate residuals and fitted values to bring back into R
            resid[i] <- y[i] - fitted_est[i]
            fitted_est[i] <- p[i] * s_size[i]
      }

      # Priors
      FLATTEN_FACTOR <- 4

      intercept ~ dnorm(0, 10^-6)
      B_doorknock ~ dnorm(0.7269,(1/(0.1780*FLATTEN_FACTOR))^2)
      B_SOGS ~ dnorm(-0.5324, (1/(0.1395*FLATTEN_FACTOR))^2)
```

```
    B_freq_thresh_m ~ dnorm(-0.1447, (1/(0.0579*FLATTEN_FACTOR))^2)
    B_freq_thresh_f ~ dnorm( -0.3809, (1/(0.0559*FLATTEN_FACTOR))^2)
    B_freq_thresh_w ~ dnorm(-0.5608, (1/(0.0610*FLATTEN_FACTOR))^2)
    B_Years ~ dnorm(0, 10^-6)
    B_Exp_EGM_pc_hdi ~ dnorm(0, 10^-6)


    # Heterogeneity statistics
    N_params <- 8

    # Calculate tau and tau2
    prec <- 1/(tau.sq)
    tau.sq <- tau^2
    tau ~ dunif(0,10)  # Suggested by http://dx.doi.org/10.1002/sim.2112

    # Calculate I2
    # As defined on p. 1546, eq. 9 and 10 http://doi.org/10.1002/sim.1186
    H.sq <- (tau.sq + sigma.sq) / sigma.sq
    sigma.sq <- (sum(w) * (N - 1)) / (sum(w)^2 - sum(w^2))
    I.sq <- tau.sq / (tau.sq + sigma.sq)

    # R2 calc
    intercept_full ~ dnorm(0, 10^-6)
    tau_full ~ dunif(0,10)
    tau.sq_full <- tau_full^2
    prec_full <- 1/(tau.sq_full)
    R.sq <- (1 - max(min(tau.sq / tau.sq_full, 1.0), 0.0)) * 100.0
    }

# Convenience settings for runjags in R:
#monitor# intercept, B_doorknock, B_SOGS, B_Years, B_Exp_EGM_pc_hdi, B_freq_thresh_m, B_freq_thresh_f, B_freq_thresh_w,
tau, tau.sq, tau_full, tau.sq_full, I.sq, H.sq, R.sq
#modules# glm on
#response# y
#residual# resid
#fitted# fitted_est
```

```
#######################################################################################################
#######################################################################################################
#### Initial values
#######################################################################################################
#######################################################################################################

inits{
"intercept" <- -1.5
"B_doorknock" <- -1.5
"B_SOGS"  <- 1.5
"B_freq_thresh_m"  <- 0
"B_freq_thresh_f"  <- 0.5
"B_freq_thresh_w"  <- 0
"B_Years"  <- -0.5
"B_Exp_EGM_pc_hdi" <- -0.5
}

inits{
"intercept" <- -0.5
"B_doorknock" <- -0.5
"B_SOGS"  <- -1.5
"B_freq_thresh_m"  <- 0
"B_freq_thresh_f"  <- 0.5
"B_freq_thresh_w"  <- 0
"B_Years"  <- 1.5
"B_Exp_EGM_pc_hdi" <- 1.5

}

inits{
"intercept" <- 0.5
"B_doorknock" <- -0.5
"B_SOGS"  <- 1.5
"B_freq_thresh_m"  <- 0
"B_freq_thresh_f"  <- 0.5
"B_freq_thresh_w"  <- 0
"B_Years"  <- 1.5
"B_Exp_EGM_pc_hdi" <- -1.5
}
```

```
inits{
"intercept" <- 1.5
"B_doorknock" <- -1.5
"B_SOGS"  <-  -0.5
"B_freq_thresh_m"  <- 0
"B_freq_thresh_f"  <- 1.5
"B_freq_thresh_w"  <- 0
"B_Years"  <- -0.5
"B_Exp_EGM_pc_hdi" <- 0.5
}
```

Listing 6: Random effects meta-analysis of the prevalence of moderate risk problem gambling, with moderators and fixed priors

```
model
      {

         for(i in 1:N) {
            y[i] ~ dbin(p[i], s_size[i])
            logit(p[i]) <- intercept +
                  B_doorknock * doorknock[i] +
                  B_SOGS * SOGS[i] +
                  B_freq_thresh_m * freq_thresh_m[i] +
                  B_freq_thresh_f * freq_thresh_f[i] +
                  B_freq_thresh_w * freq_thresh_w[i] +
                  B_Years * Years_before_2016[i] +
                  B_Exp_EGM_pc_hdi * Exp_TG_EGM_pc_hdi[i] +
                  theta[i]

            theta[i] ~ dnorm(0, prec)

            # Inverse variance weights for I2
            w[i] <- 1 / (1/y[i] + 1/(s_size[i] - y[i]))

            # Repeat with no covariates for R2 calc
            y_full[i] ~ dbin(p_full[i], s_size[i])
            logit(p_full[i]) <- intercept_full + theta_full[i]
            theta_full[i] ~ dnorm(0, prec_full)

            # calculate residuals and fitted values to bring back into R
            resid[i] <- y[i] - fitted_est[i]
            fitted_est[i] <- p[i] * s_size[i]
      }

      # Priors
      intercept ~ dnorm(0, 10^-6)
      B_doorknock ~ dnorm(0.7269, 10^24)
      B_SOGS ~ dnorm(-0.5324, 10^24)
      B_freq_thresh_m ~ dnorm(-0.1447, 10^24)
      B_freq_thresh_f ~ dnorm(-0.3809, 10^24)
```

```
    B_freq_thresh_w ~ dnorm(-0.5608, 10^24)
    B_Years ~ dnorm(0, 10^-6)
    B_Exp_EGM_pc_hdi ~ dnorm(0, 10^-6)


    # Heterogeneity statistics
    N_params <- 8

    # Calculate tau and tau2
    prec <- 1/(tau.sq)
    tau.sq <- tau^2
    tau ~ dunif(0,10)  # Suggested by http://dx.doi.org/10.1002/sim.2112

    # Calculate I2
    # As defined on p. 1546, eq. 9 and 10 in http://doi.org/10.1002/sim.1186
    H.sq <- (tau.sq + sigma.sq) / sigma.sq
    sigma.sq <- (sum(w) * (N - 1)) / (sum(w)^2 - sum(w^2))
    I.sq <- tau.sq / (tau.sq + sigma.sq)

    # R2 calc
    intercept_full ~ dnorm(0, 10^-6)
    tau_full ~ dunif(0,10)
    tau.sq_full <- tau_full^2
    prec_full <- 1/(tau.sq_full)
    R.sq <- (1 - max(min(tau.sq / tau.sq_full, 1.0), 0.0)) * 100.0
    }


# Convenience settings for runjags in R:
#monitor# intercept, B_doorknock, B_SOGS, B_Years, B_Exp_EGM_pc_hdi, B_freq_thresh_m, B_freq_thresh_f, B_freq_thresh_w,
tau, tau.sq, tau_full, tau.sq_full, I.sq, H.sq, R.sq
#modules# glm on
#response# y
#residual# resid
#fitted# fitted_est


##########################################################################################################
##########################################################################################################
```

```
#### Initial values
######################################################################################################
######################################################################################################

inits{
"intercept" <- -1.5
"B_doorknock" <- -1.5
"B_SOGS"  <- 1.5
"B_freq_thresh_m"  <- 0
"B_freq_thresh_f"  <- 0.5
"B_freq_thresh_w"  <- 0
"B_Years"  <- -0.5
"B_Exp_EGM_pc_hdi" <- -0.5
}

inits{
"intercept" <- -0.5
"B_doorknock" <- -0.5
"B_SOGS"  <- -1.5
"B_freq_thresh_m"  <- 0
"B_freq_thresh_f"  <- 0.5
"B_freq_thresh_w"  <- 0
"B_Years"  <- 1.5
"B_Exp_EGM_pc_hdi" <- 1.5

}

inits{
"intercept" <- 0.5
"B_doorknock" <- -0.5
"B_SOGS"  <- 1.5
"B_freq_thresh_m"  <- 0
"B_freq_thresh_f"  <- 0.5
"B_freq_thresh_w"  <- 0
"B_Years"  <- 1.5
"B_Exp_EGM_pc_hdi" <- -1.5
}

inits{
```

```
"intercept" <- 1.5
"B_doorknock" <- -1.5
"B_SOGS"  <-  -0.5
"B_freq_thresh_m"  <- 0
"B_freq_thresh_f"  <- 1.5
"B_freq_thresh_w"  <- 0
"B_Years"  <- -0.5
"B_Exp_EGM_pc_hdi" <- 0.5
}
```
