# Supplementary material for: A meta-regression analysis of 41 Australian problem gambling prevalence estimates and their relationship to total spending on electronic gaming machines
Source: BMC Public Health. 2017 May 23;17:495. doi: 10.1186/s12889-017-4413-6 (PMC5442595; doi:10.1186/s12889-017-4413-6)
Supplement: Supplementary file 2 — Meta-analyses of previous studies of methodological variations. An unpublished manuscript which seeks to estimate the average impact of the following methodological variations of prevalence estimates: (a) choice of problem gambling screen, (b) survey administration mode, or (c) choice of frequency threshold. (PDF 902 kb) [file 12889_2017_4413_MOESM2_ESM.pdf]

Additional file 2: Meta-analyses of previous studies of methodological variations

A meta-regression analysis of 41 Australian problem gambling prevalence estimates and their relationship to total spending on electronic gaming machines

Authors: Francis Markham *, Martin Young, Bruce Doran and Mark Sugden

Introduction

Many national and subnational jurisdictions undertake problem gambling prevalence studies to estimate the number of problem gamblers in the adult population. Indeed, over 200 problem gambling prevalence studies were conducted between 1975 and 2012 (Williams, Volberg, and Stevens, 2012), a number that continues to grow as some jurisdictions conduct their first ever problem gambling prevalence studies and others repeat the exercise in order to monitor change over time.

As Markham and Young (2016) remark, these studies have at least three objectives:

1. to assess the burden of disease in a population and to assess the need for health services;
2. to compare the prevalence of disease in different populations; and
3. to examine trends in disease prevalence or severity over time.

In practice, objectives 2 and 3 generally involve the comparison of the results of different problem gambling prevalence studies, whether this is done to compare prevalence between jurisdictions or to assess change within a single jurisdiction over time. However, as many authors have noted, such comparisons are problematic due to methodological variations between problem gambling prevalence studies (e.g. Williams & Volberg, 2009, 2010; Jackson *et al.*, 2010; Sassen, Kraus, and Bühringer, 2011; Stone et al. 2014).

This is especially problematic as the impact of methodological variations on prevalence estimates is likely to be of a greater magnitude than actual variations in underlying population prevalence. For example, a relative increase of 10% in the true prevalence of problem gambling is likely to be large enough to be policy relevant. However, as Williams and Volberg (2009) show, the impact of methodological variations on prevalence estimates may be on the order of 100% in isolation, and more than 400% when combined. Therefore, the correction for any methodological variations is important when comparing the results of prevalence studies.

Objectives

This paper undertakes meta-analyses of previous studies of the methodological variations among prevalence studies to estimate the magnitude of the corrections that must be made prior to comparing problem gambling prevalence studies. Specifically, this paper seeks to estimate the average impact of the following methodological variations of prevalence estimates:

- choice of problem gambling screen: whether problem gambling was assessed using the South Oaks Gambling Screen (SOGS) or the Problem Gambling Severity Index (PGSI)
- administration mode: whether the questionnaire was administered by telephone or face-to-face
- frequency threshold: if the problem gambling screen was administered to everyone, only to those who gambled in the last twelve months, only those who gambled in the last month, or only those who gambled in the last week.

For each of these three methodological variations, this study aims to:

1. Identify previous research on these topics

2. Summarise previous estimates of these methodological variations on problem gambling prevalence estimates

3. Describe the heterogeneity of among previous estimates

Methods

Random effects meta-analytical approach was taken to answer these research questions. Three separate analyses were undertaken in order to identify and summarise previous research on these three methodological variations in problem gambling prevalence studies.

Problem gambling screen search strategy

Studies were eligible for inclusion in this meta-analysis if they were primary studies compared and reported problem gambling prevalence estimates using both the PGSI and the SOGS. This comparison could either take place either by administering both screens to the same respondents, or by administering different screens to two random subsamples of the same sample. Studies were excluded if they applied to different time periods (i.e. if they administered the PGSI for the period of the last twelve months and SOGS over the lifetime).

Studies were identified by searching Scopus, the Web of Knowledge and by examining Williams, Volberg and Stevens' (2012) listing of problem gambling prevalence studies.

The following search terms were used in online databases:

Web of Science

```
TOPIC: (sogs AND pgsi)
Timespan: All years. Indexes: SCI-EXPANDED, SSCI, A&HCI, CPCI-S, CPCI-SSH,
ESCI, CCR-EXPANDED, IC.
```

Scopus

```
TITLE-ABS-KEY ( sogs  AND  pgsi )
```

Using the listing of problem gambling prevalence studies from Williams, Volberg and Stevens' (2012), studies were identified that administered both the SOGS and PGSI.

Studies were screened by FM to ensure that the eligibility criteria were met, and data extracted into a spreadsheet. Where prevalence of problem gambling was reported as a percentage, these estimates were converted to a count of cases. The following data items were extracted for each study:

1. Whether both the SOGS and the PGSI were administered to respondents, or whether different subgroups of respondents were administered different screens
2. The count of respondents endorsing 5 more SOGS items
3. The denominator for this count
4. The count of respondents scoring 8 more on the PGSI
5. The denominator for this count
6. The count of respondents endorsing 3-4 SOGS items
7. The denominator for this count
8. The count of respondents scoring 3-7 on the PGSI
9. The denominator for this count

Administration mode search strategy

Studies were eligible for inclusion in this meta-analysis if they were primary studies which compared and reported problem gambling prevalence estimates from surveys administered by telephone and face-to-face. Only experiments which administered a common survey instrument to a single sample frame, using random selection to assign respondents to an administration mode were eligible for inclusion. Studies which compared telephone surveys with postal surveys were ineligible.

Studies were identified by searching Scopus and the Web of Knowledge and by examining Williams, Volberg and Stevens' (2012) listing of problem gambling prevalence studies.

The following search terms were used in online databases:

Web of Science

```
TOPIC:
(gambling OR "problem gambling" OR "pathological gambling" OR pgsi OR cpgi
OR sogs) AND
TOPIC: (( "administration mode" OR "administration format" OR "face-to-
face"))
Timespan: All years. Indexes: SCI-EXPANDED, SSCI, A&HCI, CPCI-S, CPCI-SSH,
ESCI, CCR-EXPANDED, IC.
```

Scopus

```
( TITLE-ABS-KEY ( gambling  OR  "problem gambling"  OR  "pathological
gambling"  OR  pgsi  OR  cpgi  OR  sogs )  AND  TITLE-ABS-KEY (
"administration mode"  OR  "administration format"  OR  "face-to-face" ) )
```

The full text of relevant studies was read for further references.

Studies were screened by FM to ensure that the eligibility criteria were met, and data extracted into a spreadsheet. Where prevalence of problem gambling was reported as a percentage, these estimates were converted to a count of cases. If results were reported with population weights applied and without population weights applied, both data values were extracted. The following data items were extracted for each study:

1. The count of respondents classified as problem gamblers (PGSI ≥ 8 or SOGS ≥ 5) in the telephone survey
2. The denominator for this count
3. The count of respondents classified as problem gamblers (PGSI ≥ 8 or SOGS ≥ 5) in the face-to-face survey
4. The denominator for this count
5. The count of respondents classified as moderate risk problem gamblers (PGSI 3-7 or SOGS 3-4) in the telephone survey
6. The denominator for this count
7. The count of respondents classified as moderate risk problem gamblers (PGSI 3-7 or SOGS 3-4) in the face-to-face survey
8. The denominator for this count

Frequency threshold search strategy

Studies were eligible for inclusion in this meta-analysis if they were primary studies compared and reported problem gambling prevalence estimates made on the basis of different frequency thresholds for administering the problem gambling screen. This comparison could either take place either by administering both screens to the same respondents, or by administering different screens to two random subsamples of the same sample.

Studies were identified by searching Scopus, the Web of Knowledge and by examining Williams, Volberg and Stevens' (2012) listing of problem gambling prevalence studies. All studies in Williams, Volberg and Stevens' (2012) list were eligible if a) they were not the first problem gambling prevalence study in that jurisdiction, b) the previous study in that jurisdiction only administered the problem gambling screen to those respondents who met a gambling frequency threshold, and c) that study used no frequency threshold, or a lower frequency threshold. The logic behind these criteria is that in eligibility studies, a valid comparison of problem gambling prevalence with a past estimate in the same jurisdiction requires re-estimation using a different frequency threshold.

The following search terms were used in online databases:

Web of Science

```
TOPIC: (("frequency threshold" OR "sub-sample" OR "exclusion criteria" OR
"regular gamblers" OR "non-regular gamblers" OR "frequent gamblers" OR
"infrequent gamblers")) AND TOPIC: ((gambling OR "problem gambling" OR
"pathological gambling" OR pgsi OR cpgi OR sogs))
Timespan: All years. Indexes: SCI-EXPANDED, SSCI, A&HCI, CPCI-S, CPCI-SSH,
ESCI, CCR-EXPANDED, IC.
```

Scopus

```
( TITLE-ABS-KEY ( "frequency threshold"  OR  "sub-sample"  OR  "exclusion
criteria"  OR  "regular gamblers"  OR  "non-regular gamblers"  OR
"frequent gamblers"  OR  "infrequent gamblers" )  AND  TITLE-ABS-KEY (
gambling  OR  "problem gambling"  OR  "pathological gambling"  OR  pgsi  OR
cpgi  OR  sogs ) )
```

Using the exclusion criteria described above and the listing of problem gambling prevalence studies from Williams, Volberg and Stevens' (2012), studies were identified that reported problem gambling prevalence estimates based on two different frequency thresholds.

Studies were screened by FM to ensure that the eligibility criteria were met, and data extracted into a spreadsheet. Where prevalence of problem gambling was reported as a percentage, these estimates were converted to a count of cases. Where studies included other exclusion criteria in addition to a frequency threshold (e.g. they ignored lottery gambling when selecting a subsample) these other criteria were ignored. Studies that administered the problem gambling screen only to those who had gambled at least once in the past year or in their lifetime were grouped with studies that administered the screen to all respondents. The following data items were extracted for each study:

1. Whether the problem gambling screen was the SOGS or the PGSI
2. The count of problem gamblers (PGSI ≥ 8 or SOGS ≥ 5) when the screen was administered only to those who gambled weekly or more frequently
3. The denominator for this count
4. The count of moderate risk problem gamblers (PGSI 3-7 or SOGS 3-4) when the screen was administered only to those who gambled weekly or more frequently
5. The denominator for this count
6. The count of problem gamblers (PGSI ≥ 8 or SOGS ≥ 5) when the screen was administered only to those who gambled fortnightly or more frequently
7. The denominator for this count
8. The count of moderate risk problem gamblers (PGSI 3-7 or SOGS 3-4) when the screen was administered only to those who gambled fortnightly or more frequently

9. The denominator for this count

10. The count of problem gamblers (PGSI ≥ 8 or SOGS ≥ 5) when the screen was administered only to those who gambled monthly or more frequently

11. The denominator for this count

12. The count of moderate risk problem gamblers (PGSI 3-7 or SOGS 3-4) when the screen was administered only to those who gambled monthly or more frequently

13. The denominator for this count

14. The count of problem gamblers (PGSI ≥ 8 or SOGS ≥ 5) when the screen was administered to everyone or those who had ever gambled or those who had gambled in the past year

15. The denominator for this count

16. The count of moderate risk problem gamblers (PGSI 3-7 or SOGS 3-4) when the screen was administered to everyone or those who had ever gambled or those who had gambled in the past year

17. The denominator for this count

Statistical analysis

The principal summary measures are the ratios of prevalence estimates in the contrasting conditions. These are calculated as a risk ratio would be calculated in a conventional meta-analysis.

As it was anticipated that prevalence ratios would exhibit substantial heterogeneity, it was decided to use a random effects meta-analysis model. Each meta-analysis was repeated twice, once for problem gamblers (PGSI ≥ 8 or SOGS ≥ 5) and once for moderate risk problem gamblers (PGSI 3-7 or SOGS 3-4). As well as estimating mean prevalence ratios and their 95% confidence intervals, 95% prediction intervals were calculated in order to estimate the range of values expected in future studies of this type. τ and I^2, measures of the heterogeneity of studies were also calculated and reported in each meta-analysis. No assessment of the risk of bias was made for individual studies. No review protocol was registered for this study.

Results

Problem gambling screen

A total of 237 studies were screened, yielding 12 studies that met the eligibility criteria (see Fig. 1). Characteristics of individual studies included are listed in Table 1.

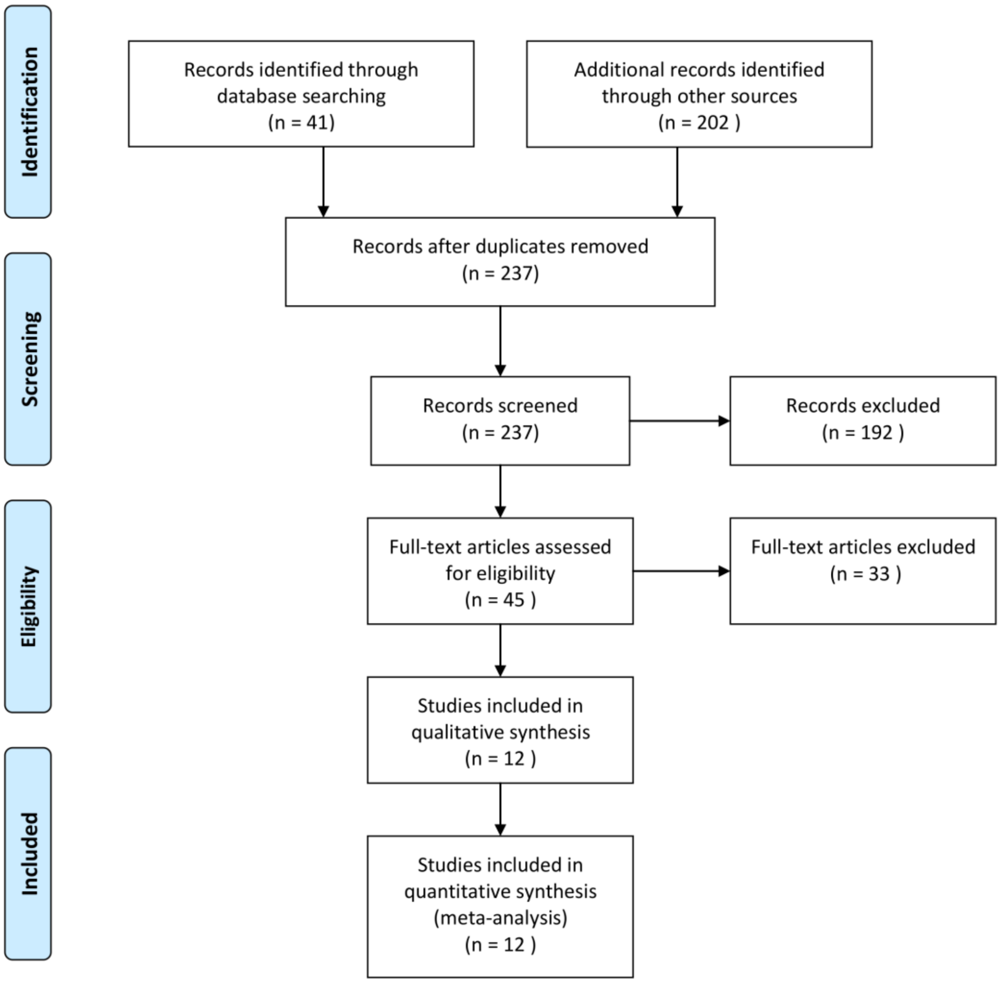

Fig. 1: Literature search flow diagram for problem gambling screen meta-analysis

Table 1: Studies of problem gambling screen effects from which data were extracted

Study	Same respondents	SOGS 5+	SOGS *n*	PGSI 8+	PGSI *n*	SOGS 3-4	PGSI 3-7
NT 2005	TRUE	54	369	38	369	50	68
VIC 2003	FALSE	26	143	22	141	22	21
BPDS 2010	TRUE	32	2193	13	2193	25	77
IODS 2010	TRUE	664	5078	467	5079	730	1247
Ferris and Wynne 2001	TRUE	41	3120	28	3120	41	74
British Columbia 2002	TRUE	23	2134	10	2500	58	105
Manitoba 2001	TRUE	72	3119	34	3119	NA	72
Italy 2008	TRUE	69	1987	73	1987	64	233
Sweden 2009	TRUE	120	15000	45	15000	180	285

Hungary 2013	TRUE	78	586	37	586	75	68
Quebec 2002	FALSE	41	4603	30	4225	41	42
Turner et al. 2009	TRUE	33	254	24	254	12	40

As Fig. 2 shows, the mean ratio of problem gambling prevalence estimates made with SOGS ≥ 5 and PGSI ≥ 8 was 1.6 (95% C.I. 1.3, 2.0), meaning that studies conducted using SOGS identified an average of 1.6 times more problem gamblers. The 95% prediction interval around this estimate was 0.9 - 2.8. A τ value of 0.27 (95% C.I. 0.12, 0.54) was recorded, and an I^2 of 69% (95% C.I. 32%, 90%), a moderate degree of heterogeneity.

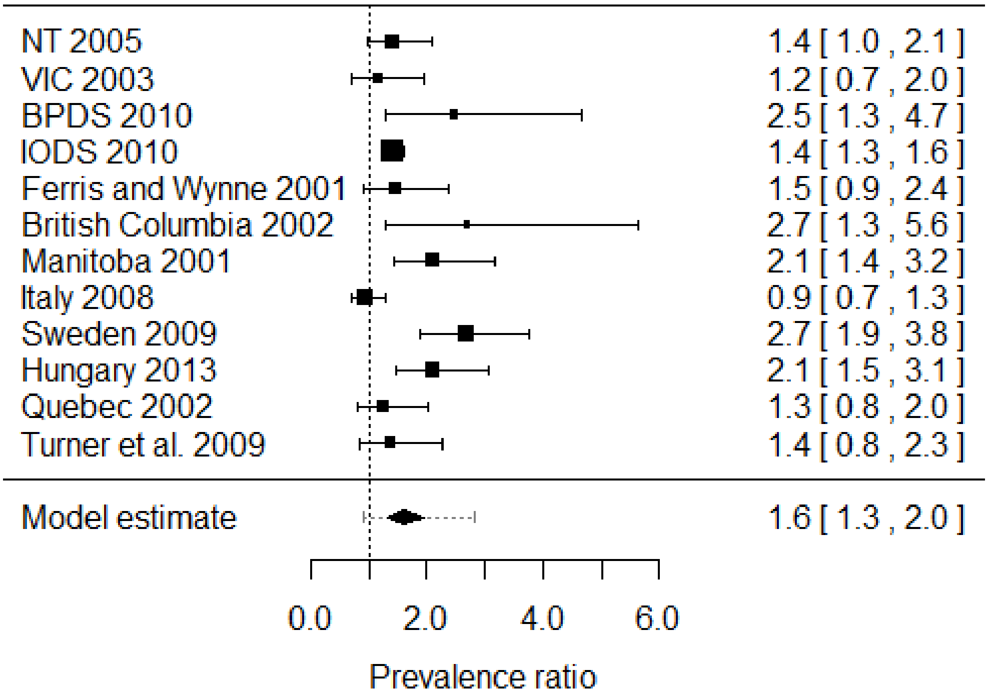

Figure 2: SOGS 5+ to PGSI 8+ prevalence ratios and model estimate. Dotted line indicates 95% prediction interval.

The prevalence ratio is quite different for moderate risk problem gambling (Fig. 3). The mean ratio of the prevalence of SOGS 3-4 and PGSI 3-7 was 0.6 (95% C.I. 0.4, 0.8), meaning that studies conducted using SOGS identified an average of 0.6 times fewer people. The 95% prediction interval around this estimate was 0.2 - 1.4. A τ value of 0.42 (95% C.I. 0.27, 0.82) was recorded, and an I^2 of 91% (95% C.I. 80%, 97%), a very high degree of heterogeneity.

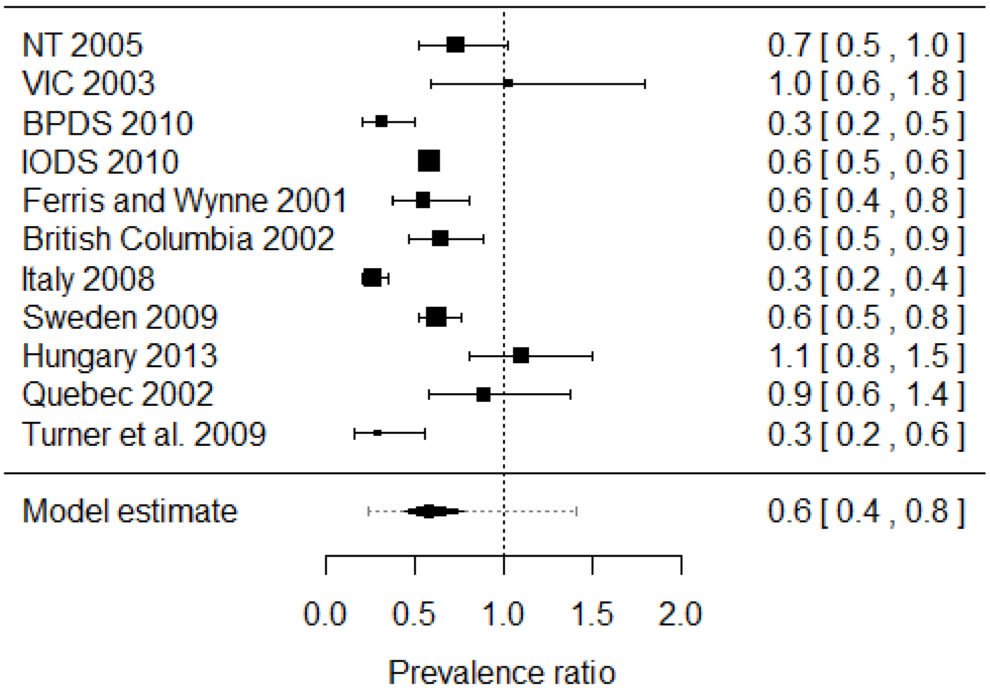

Fig. 3: SOGS 3-4 to PGSI 3-7 prevalence ratios and model estimate. Dotted line indicates 95% prediction interval.

Administration mode

Although 81 studies were screened, only a single study that met the eligibility criteria was identified (see Fig. 4). That study published two estimates of the effect of administration, one with population weights applied and another unweighted estimate. The results of these two estimates are listed in Table 2.

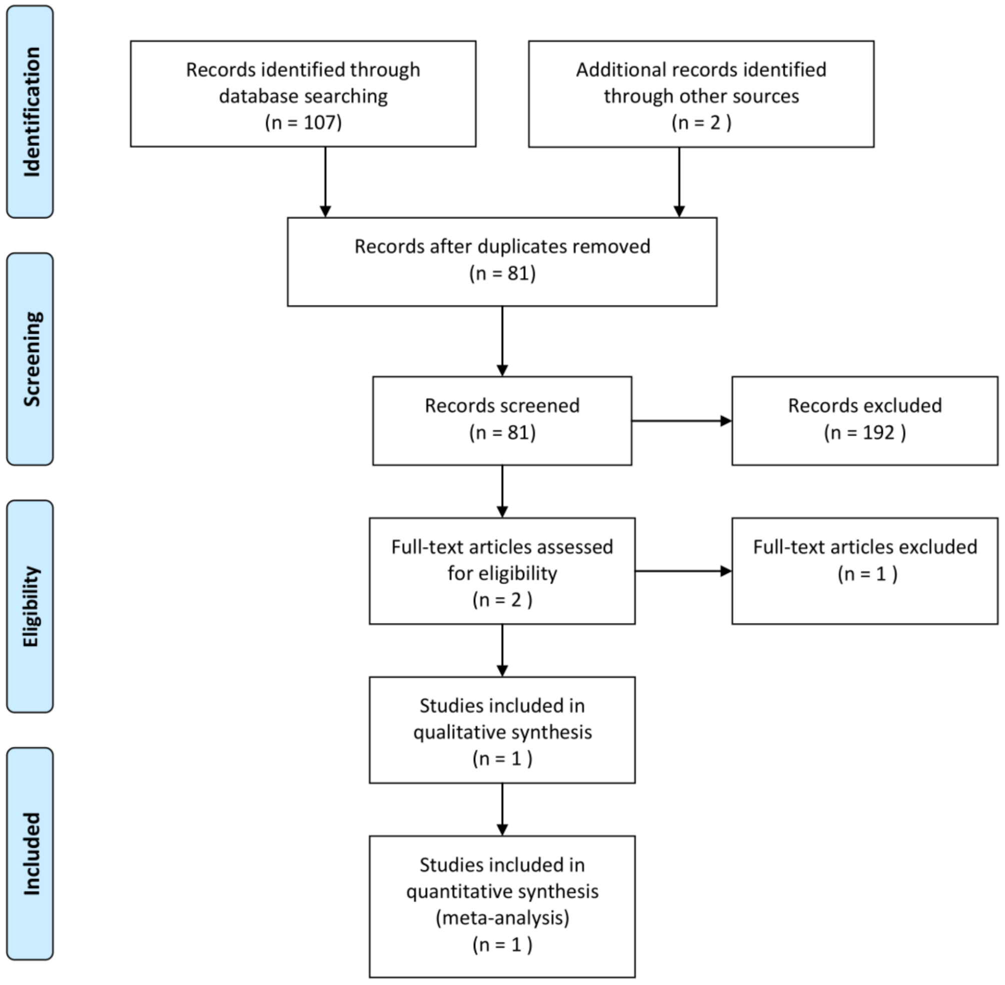

Fig. 4: Literature search flow diagram for administration mode meta-analysis

Table 2: Estimates from which data were extracted

	Problem gambling				Moderate risk problem gambling			
Study	Telephone	Telephone *n*	Face to face	Face to face *n*	Telephone	Telephone *n*	Face to face	Face to face *n*
W & V 2009 unweighted	6	1513	7	1515	22	1513	54	1515
W & V 2009 weighted	6	1518	6	1528	24	1518	42	1528

As Fig. 5 shows, the mean ratio of problem gambling prevalence estimates administered by doorknock compared to telephone was 1.1 (95% C.I. 0.5, 2.4), meaning that studies administered face-to-face identified an average of 1.1 times more problem gamblers. As only two reports of a single study were identified, heterogeneity was unable to be estimated.

Results were much more extreme for moderate risk problem gamblers (Fig. 6). The mean ratio of moderate risk prevalence estimates administered by doorknock compared to telephone was 2.1 (95% C.I. 1.5, 2.9), meaning that studies administered face-to-face identified an average of 2.1 times more moderate risk gamblers.

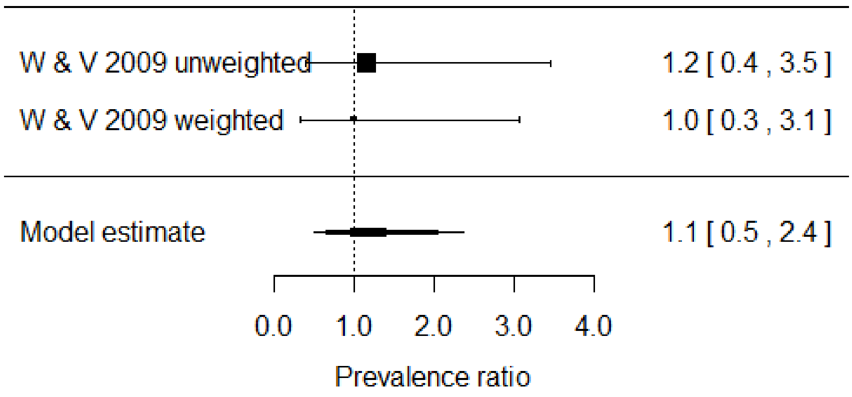

Figure 5: Doorknock administered to telephone administered prevalence ratios and model estimate for PGSI >= 8.

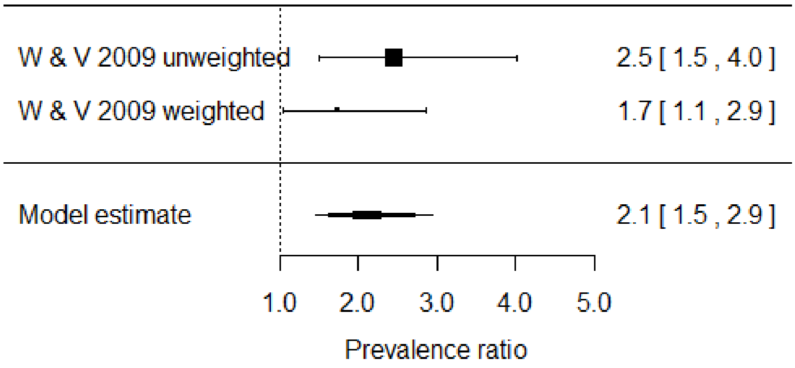

Figure 6: Doorknock administered to telephone administered prevalence ratios and model estimate for PGSI 3-7.

Frequency threshold

A total of 322 studies were screened, yielding 5 studies that met the eligibility criteria (see Fig. 7). Characteristics of individual studies included are listed in Table 3.

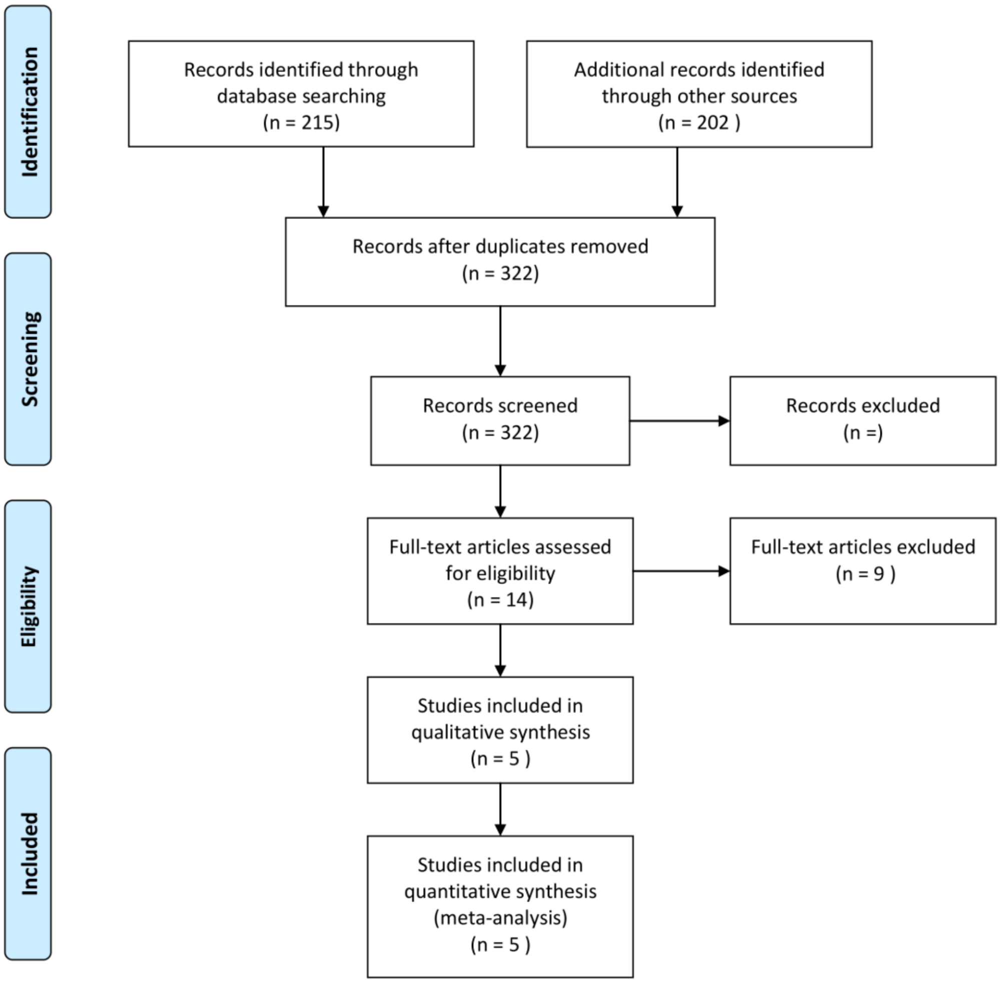

Fig. 7: Literature search flow diagram for frequency threshold meta-analysis

Table 3: Estimates from which data were extracted

		Weekly threshold			Fortnightly threshold			Monthly threshold			Annual or no threshold		
Study	Screen	Problem gamblers	Moderate risk problem gamblers	*n*	Problem gamblers	Moderate risk problem gamblers	*n*	Problem gamblers	Moderate risk problem gamblers	*n*	Problem gamblers	Moderate risk problem gamblers	*n*
NSW 2011	PGSI	40	150	10000							80	290	10000
SA 2012	PGSI				49	143	9246				55	231	9246
TAS 1996	SOGS	27	42	1211							35	69	1211
TAS 2011	PGSI	17	30	4303							30	77	4303
Jackson, Wynne et al 2009 (VIC 2007)	PGSI	18	39	1488							28	56	1488
Stone et al. - VIC 2008	PGSI	81	195	15000	93	233	15000	101	300	15000	105	350	15000
Stone et al. - Swelogs 2008	PGSI	36	185	15000	47	213	15000	47	246	15000	48	281	15000

As Fig. 8 shows, the mean ratio of problem gambling prevalence estimates made with a weekly frequency threshold compared to an annual frequency threshold was 0.7 (95% C.I. 0.6, 0.8), meaning that studies conducted using a weekly frequency threshold identified an average of 0.7 times fewer problem gamblers. The 95% prediction interval around this estimate was 0.5 - 0.8 A τ value of 0.06 (95% C.I. 0.00, 0.39) was recorded, and an I^2 of 7% (95% C.I. 0%, 76%), a very small degree of heterogeneity.

Less of an effect can be detected for fortnightly frequency thresholds, perhaps due to the smaller number of studies that have analysed them. Fig. 9 shows that the mean ratio of problem gambling prevalence estimates made with a fortnightly frequency threshold compared to an annual frequency threshold was 0.9 (95% C.I. 0.7, 1.1), meaning that studies conducted using a fortnightly frequency threshold identified an average of 0.9 times fewer problem gamblers. This is not significantly different to no effect. Heterogeneity could not be estimated for fortnightly thresholds.

A similar result was found for month frequency thresholds. Fig. 10 shows just two studies of these thresholds, with a mean prevalence ratio of 1.0 (95% C.I. 0.8, 1.2). This is not significantly different to no effect. Heterogeneity could not be estimated for fortnightly thresholds.

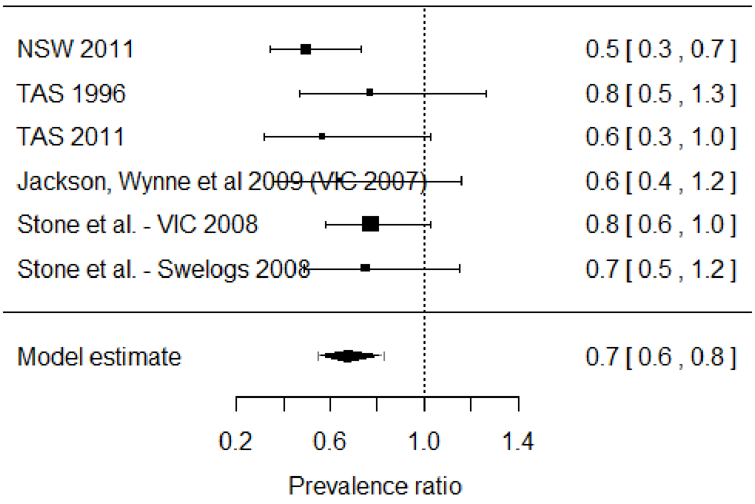

Figure 8: Ratio of problem gambling prevalence estimates for weekly frequency thresholds compared to annual frequency thresholds. Dotted line indicates 95% prediction interval.

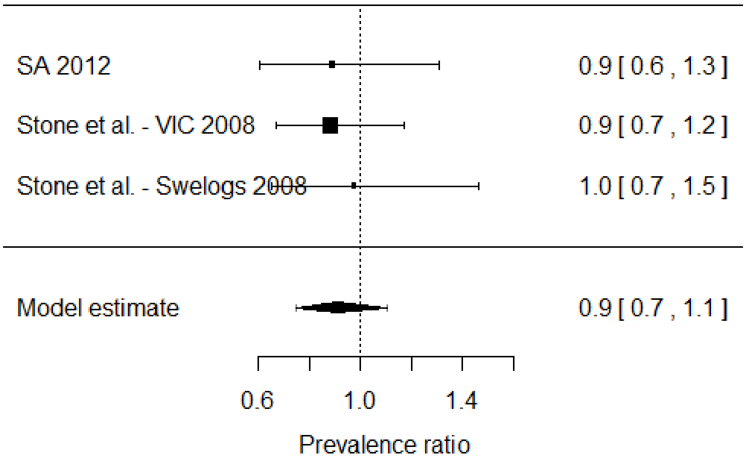

Figure 9: Ratio of problem gambling prevalence estimates for fortnightly frequency thresholds compared to annual frequency thresholds. Dotted line indicates 95% prediction interval.

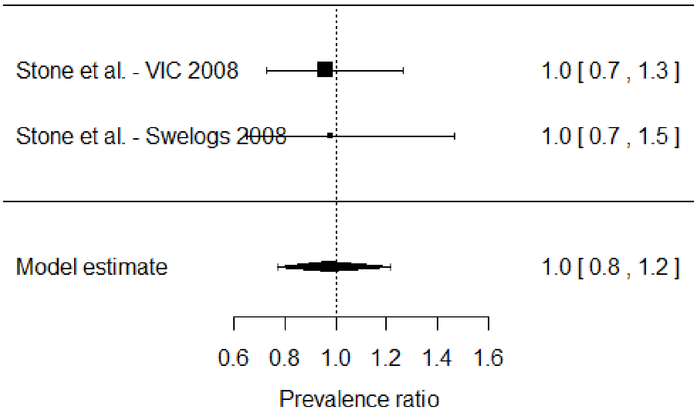

Figure 10: Ratio of problem gambling prevalence estimates for monthly frequency thresholds compared to annual frequency thresholds. Dotted line indicates 95% prediction interval.

Frequency thresholds appear to have a stronger effect on moderate risk problem gambling (see Figs. 11-13). For moderate risk problem gamblers (PGSI 3-7 and SOGS 3-4), the effect of weekly, fortnightly and monthly frequency thresholds were estimated in terms of prevalence ratios as 0.6 (95% C.I. 0.5, 0.6), 0.7 (95% C.I. 0.6, 0.8) and 0.9 (95% C.I. 0.8, 1.0) respectively.

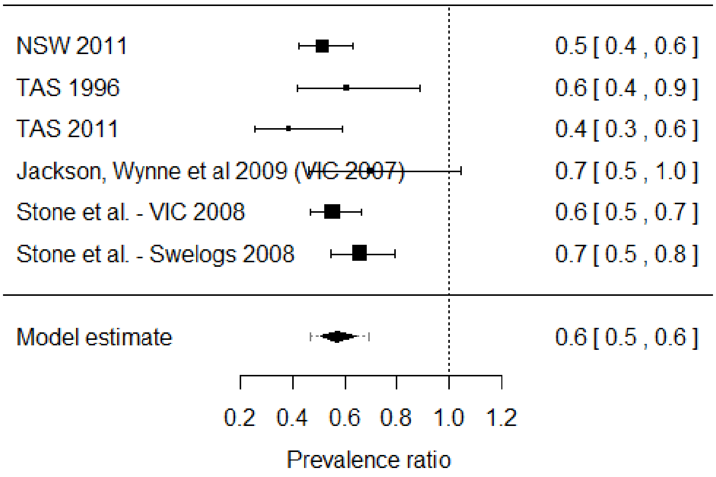

Figure 11: Ratio of moderate risk problem gambling prevalence estimates for weekly frequency thresholds compared to annual frequency thresholds. Dotted line indicates 95% prediction interval.

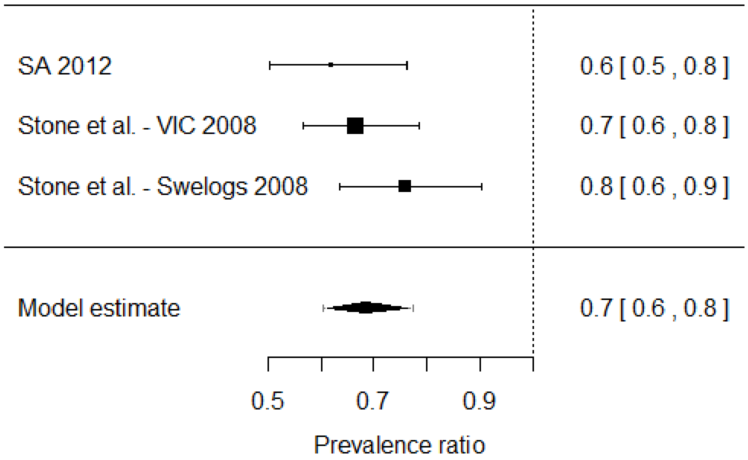

Figure 12: Ratio of moderate risk problem gambling prevalence estimates for fortnightly frequency thresholds compared to annual frequency thresholds. Dotted line indicates 95% prediction interval.

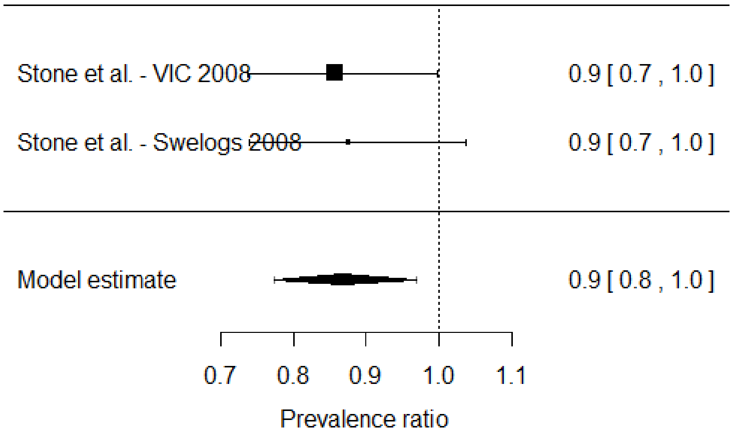

Figure 13: Ratio of moderate risk problem gambling prevalence estimates for monthly frequency thresholds compared to annual frequency thresholds. Dotted line indicates 95% prediction interval.

Discussion and conclusions

In the case of problem gambling, there is substantial evidence that screen choice has a significant impact on prevalence estimates. More precisely, estimates using SOGS ≥ 5 identify 1.6 (95% C.I. 1.3, 2.0) times more problem gamblers than studies using PGSI ≥ 8. Only a single study of the effect of administration mode on problem gambling prevalence estimates could be identified, and it found no evidence of a significant effect. Studies which administered a problem gambling screen only to weekly gamblers identified an average of 0.7 (95% C.I. 0.6, 0.8) times fewer problem gamblers. No significant effect on problem gambler prevalence was found for fortnightly or monthly thresholds, but this could be due to the small number of studies that investigated these thresholds.

For moderate risk problem gambling, defined as a SOGS score of 3-4 or a PGSI score of 3-7, results were quite different. Specifically, studies using SOGS 3-4 found 0.6 (95% C.I. 0.4, 0.8) times fewer moderate risk problem gamblers, compared with those using PGSI 3-7. While administration mode appeared to matter little for problem gambling, for moderate risk problem gambling a significant effect was found. Studies administered face-to-face identified 2.1 (95% C.I. 1.5, 2.9) times more moderate risk problem gamblers than those administered by telephone. Similarly, frequency thresholds had a greater effect on estimates of the prevalence of moderate risk problem gambling than problem gambling. Specifically, for moderate risk problem gamblers (PGSI 3-7 and SOGS 3-4), the prevalence ratios associated with weekly, fortnightly and monthly frequency thresholds were 0.6 (95% C.I. 0.5, 0.6), 0.7 (95% C.I. 0.6, 0.8) and 0.9 (95% C.I. 0.8, 1.0) respectively.

Due to the small number of studies meeting the inclusion criteria, these results should be interpreted with caution. In particular, only a single study of the effect of administration mode on prevalence estimates was found, and only two studies investigated the impact of fortnightly and monthly frequency thresholds. Furthermore, the extreme heterogeneity was evident in estimates of the effect of screen choice on the prevalence of moderate risk problem gambling, meaning that estimates of these effects vary substantially from study to study.

References

Jackson, Alun C., Harold Wynne, Nicki A. Dowling, Jane E. Tomnay, and Shane A. Thomas. 2010. "Using the CPGI to Determine Problem Gambling Prevalence in Australia: Measurement Issues." *International Journal of Mental Health and Addiction* 8 (4): 570–82. doi:10.1007/s11469-009-9238-9.

Markham, F., and Young, M. (2016) Commentary on Dowling *et al*. (2016): Is it time to stop conducting problem gambling prevalence studies?. *Addiction*, 111: 436–437. doi:10.1111/add.13216.

Sassen, Monika, Ludwig Kraus, and Gerhard Bühringer. 2011. "Differences in Pathological Gambling Prevalence Estimates: Facts or Artefacts?" *International Journal of Methods in Psychiatric Research* 20 (4): e83–e99. doi:10.1002/mpr.354.

Stone, Christine A., Ulla Romild, Max Abbott, Kristal Yeung, Rosa Billi, and Rachel Volberg. 2014. "Effects of Different Screening and Scoring Thresholds on PGSI Gambling Risk Segments." *International Journal of Mental Health and Addiction* 13 (1): 82–102. doi:10.1007/s11469-014-9515-0.

Williams, Robert J., and Rachel A. Volberg. 2010. "Best Practices in the Population Assessment of Problem Gambling," March.

Williams, Robert J., Rachel A. Volberg, and Rhys M. G. Stevens. 2012. "The Population Prevalence of Problem Gambling: Methodological Influences, Standardized Rates, Jurisdictional Differences, and Worldwide Trends." Technical Report. Ontario Problem Gambling Research Centre.

Williams, Robert J., and Rachel A. Volberg. 2009. "Impact of Survey Description, Administration Format, and Exclusionary Criteria on Population Prevalence Rates of Problem Gambling." *International Gambling Studies* 9 (2): 101–17. doi:10.1080/14459790902911653.
