# Supplementary material for: A meta-regression analysis of 41 Australian problem gambling prevalence estimates and their relationship to total spending on electronic gaming machines
Source: BMC Public Health. 2017 May 23;17:495. doi: 10.1186/s12889-017-4413-6 (PMC5442595; doi:10.1186/s12889-017-4413-6)
Supplement: Supplementary file 3 — Supplementary figures and tables. Study inclusion flow diagram; problem gambling prevalence forest plot; moderate risk problem gambling prevalence forest plot; and full bibliographic details for each study. (PDF 1173 kb) [file 12889_2017_4413_MOESM3_ESM.pdf]

Additional file 3: Supplementary figures and tables

A meta-regression analysis of 41 Australian problem gambling prevalence estimates and their relationship to total spending on electronic gaming machines

Authors: Francis Markham, Martin Young, Bruce Doran and Mark Sugden

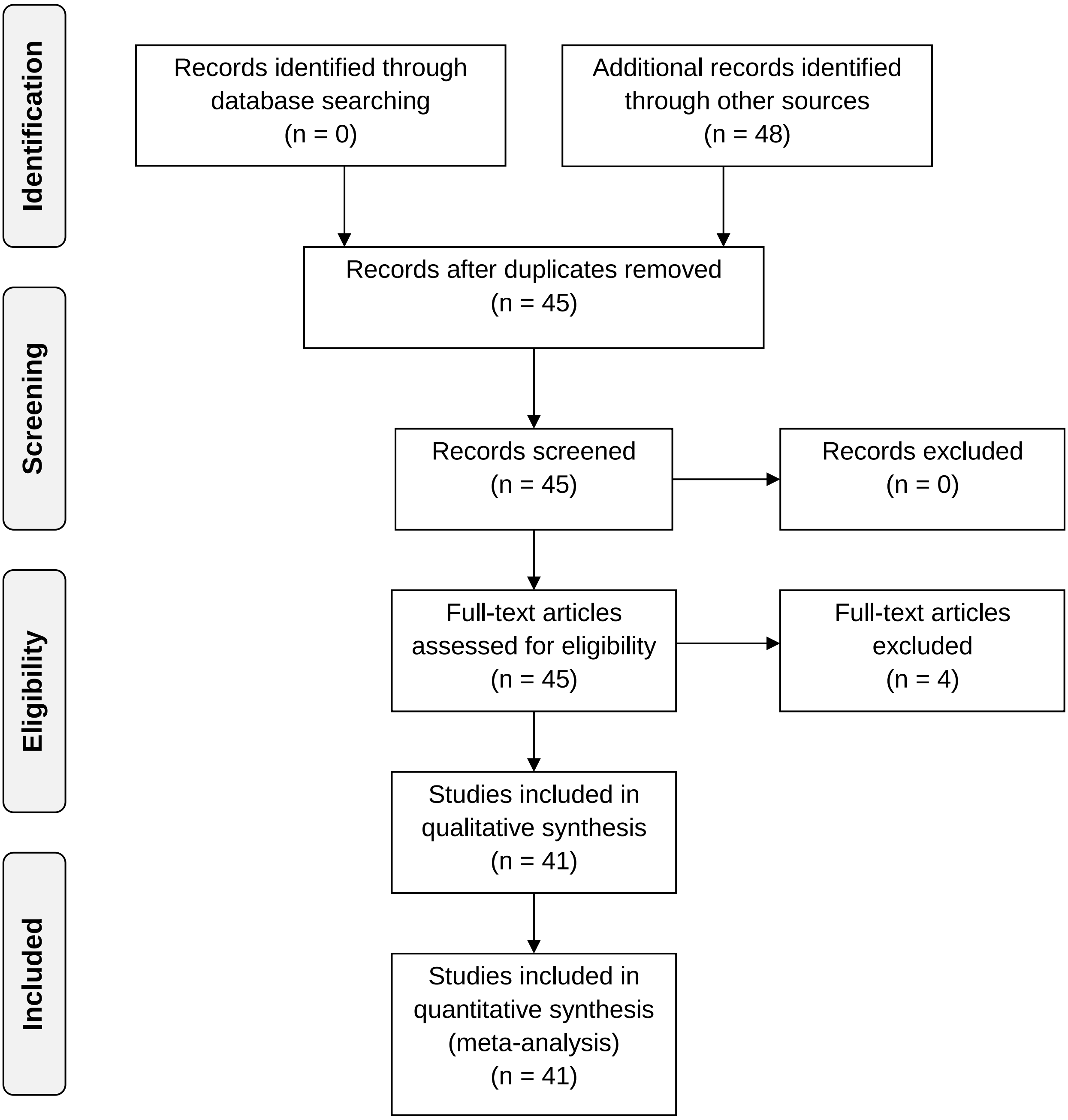

Figure S1: Prevalence study records identified, included and excluded.

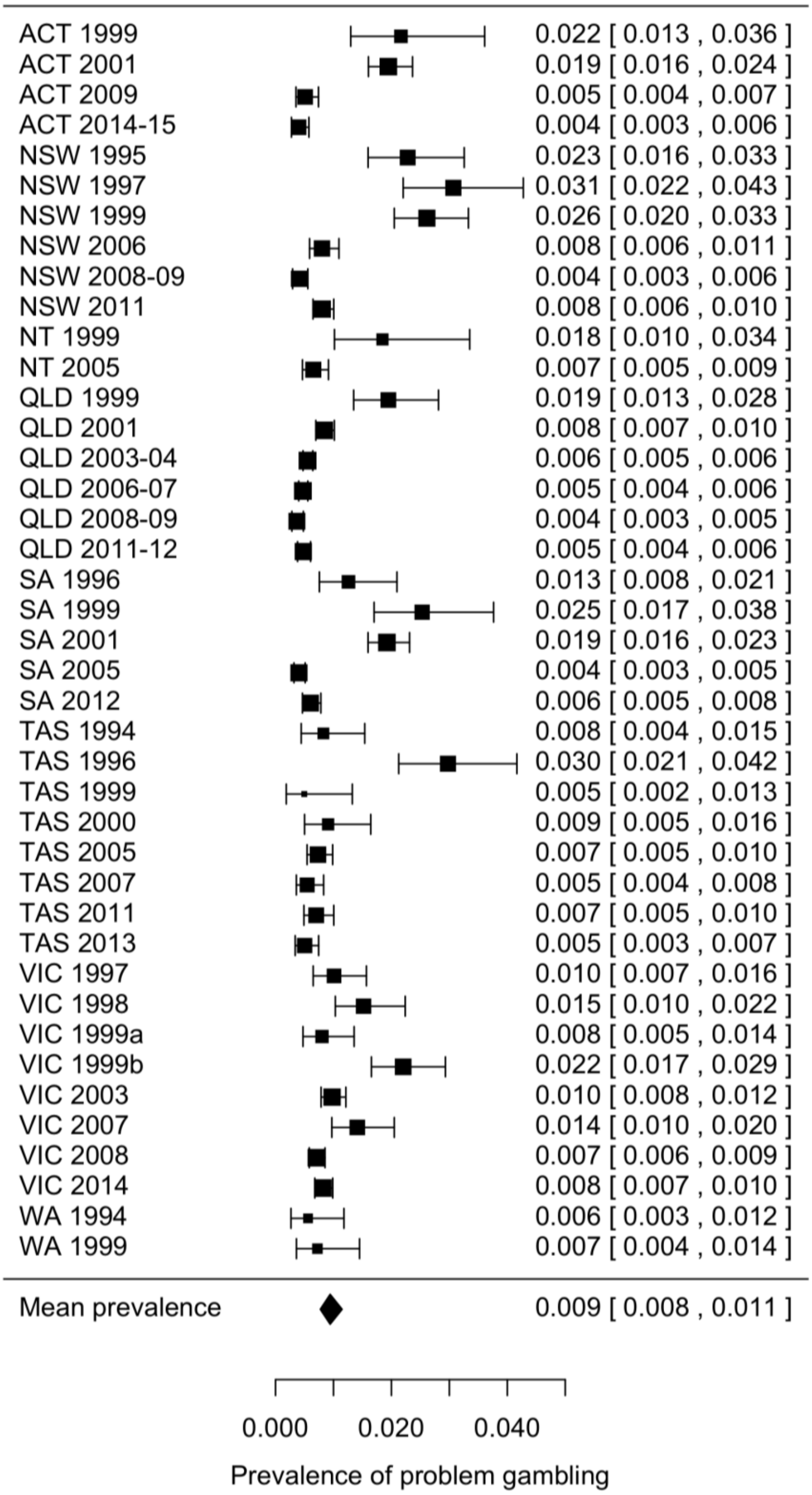

Figure S2: Problem gambling prevalence estimates in all individual studies (*n* = 41) and mean prevalence estimated by random effects meta-analysis.

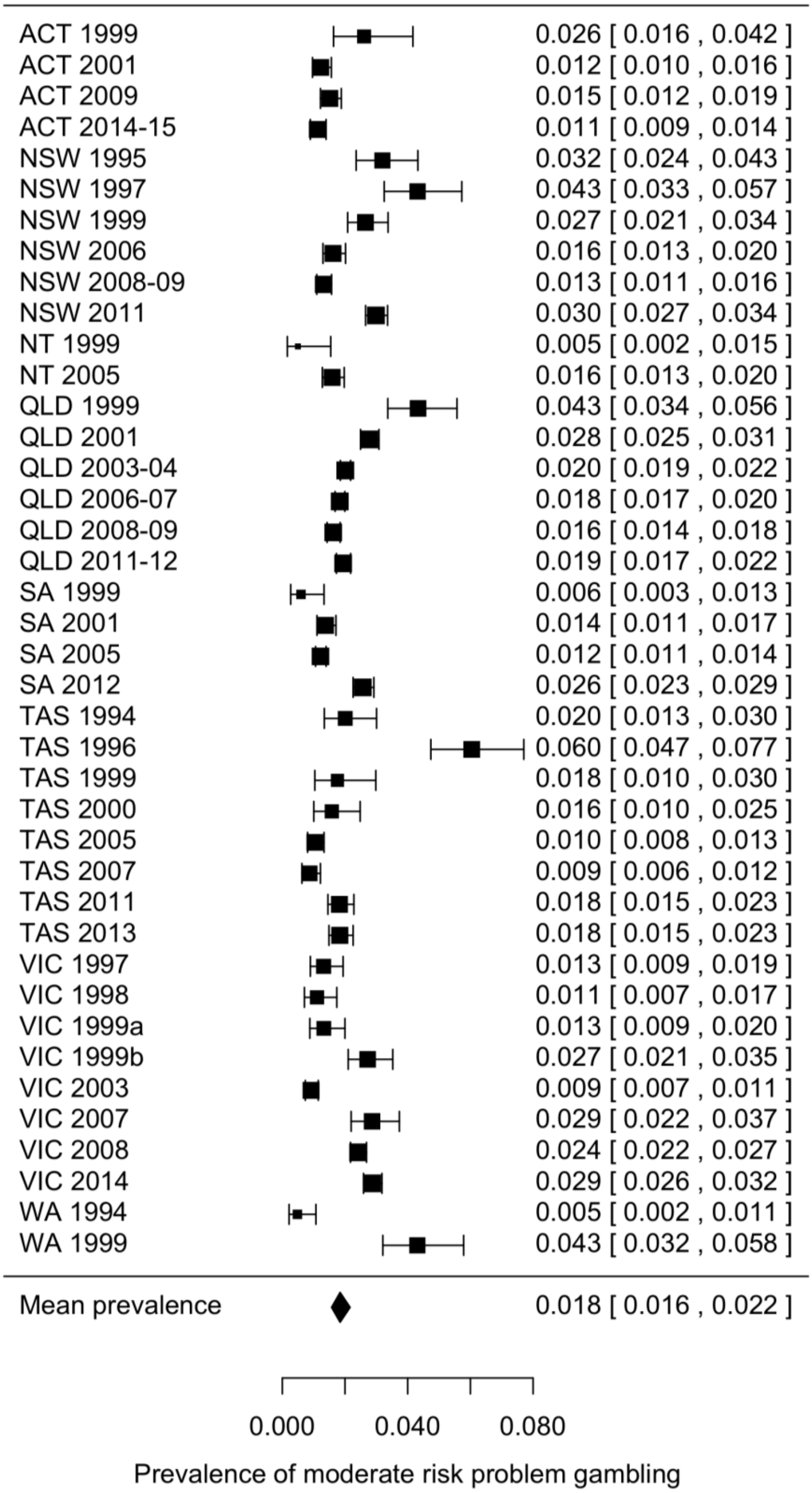

Figure S3: Moderate risk problem gambling prevalence estimates in all individual studies (*n* = 40) and mean prevalence estimated by random effects meta-analysis.

Table S1: Bibliographic details for each study

State or territory	Year	Full bibliography
ACT	1999	Productivity Commission. Australia's Gambling Industries. Report no.: 10.Canberra: Productivity Commission; 1999. Available from: http://www.pc.gov.au/inquiries/completed/gambling/report/gambling1.pdf archived at http://www.webcitation.org/6k8jP5l5E
ACT	2001	Tremayne K, Masterman-Smith H, McMillen J. Survey of the nature and extent of gambling and problem gambling in the ACT [Internet]. Sydney: Australian Institute for Gambling Research, University of Western Sydney; 2001. Available from: http://www.gamblingandracing.act.gov.au/__data/assets/pdf_file/0009/745065/Survey-of-Problem-Gambling-in-the-ACT.pdf archived at http://www.webcitation.org/6k8jsBDpr
ACT	2009	Davidson T, Rodgers B. 2009 Survey of the nature and extent of gambling, and problem gambling in the Australian Capital Territory [Internet]. Canberra: The Centre for Gambling Research, The Australian National University; 2010. Available from: http://www.problemgambling.act.gov.au/Recent%20Research/ACT%20Gambling%20Prevalence%20Study.pdf archived at http://www.webcitation.org/6k8kG4vKu
ACT	2014-15	Davidson T, Rodgers B, Taylor-Rodgers E, Suomi A, Lucas N. 2014 survey on gambling, health and wellbeing in the ACT [Internet]. Canberra: Centre for Gambling Research, The Australian National University; 2015. Available from: http://sociology.cass.anu.edu.au/sites/default/files/2014%20Survey%20on%20gambling%20health%20and%20wellbeing%20in%20the%20ACT.pdf archived at http://www.webcitation.org/6k8kVQRKs
NSW	1995	Dickerson M, Allcock C, Blaszczynski A, Williams J, Maddern R. An examination of the socio-economic effects of gambling on individuals, families and the community, including research into the costs of problem gambling in New South Wales [Internet]. Sydney: Australian Institute for Gambling Research, University of Western Sydney; 1996. Available from: https://www.liquorandgaming.justice.nsw.gov.au/Documents/gaming-and-wagering/problems-with-gambling/research/2.%20Study%202%20%E2%80%93%20An%20Examination%20of%20the%20Socio-economic%20Effects%20of%20Gambling%20on%20Individuals,%20Families%20and%20the%20Community%20-%20part%201.pdf archived at http://www.webcitation.org/6k8kzUWQP
NSW	1997	Dickerson M, Allcock C, Blaszczynski A, Maddern R, Nicholls B, Williams J. An examination of the socio-economic effects of gambling on individuals, families and the community, including research into the costs of problem gambling (study 2 update) [Internet]. Sydney: Australian Institute for Gambling Research, University of Western Sydney; 1998. Available from: https://www.liquorandgaming.justice.nsw.gov.au/Documents/gaming-and-wagering/problems-with-gambling/research/1.%20An%20Examination%20of%20the%20Socio-Economic%20Effects%20of%20Gambling%20-%20part%201.pdf and https://www.liquorandgaming.justice.nsw.gov.au/Documents/gaming-and-wagering/problems-with-gambling/research/1.%20An%20Examination%20of%20the%20Socio-Economic%20Effects%20of%20Gambling%20-%20part%202.pdf archived at http://www.webcitation.org/6k8lSm0dN and http://www.webcitation.org/6k8lSwkR0
NSW	1999	Productivity Commission. Australia's Gambling Industries. Report no.: 10.Canberra: Productivity Commission; 1999. Available from: http://www.pc.gov.au/inquiries/completed/gambling/report/gambling1.pdf archived at http://www.webcitation.org/6k8jP5l5E
NSW	2006	ACNielsen. Prevalence of gambling and problem gambling in NSW: A community survey 2006 [Internet]. ACNielsen; 2007. Available from: https://www.liquorandgaming.justice.nsw.gov.au/Documents/gaming-and-wagering/problems-with-

		gambling/research/8.%20Prevalence%20of%20Gambling%20and%20Problem%20Gambling%20in%20NSW%20%E2%80%93%20A%20Community%20Survey%202006.pdf archived at http://www.webcitation.org/6k8m1TlZC
NSW	2008-09	NSW Department of Health. Gambling Module, NSW Population Health Survey, 2008-2009 [Internet]. Sydney: NSW Department of Health; 2009. Available from: https://www.liquorandgaming.justice.nsw.gov.au/Documents/gaming-and-wagering/problems-with-gambling/research/1.%20Gambling%20Module%20NSW%20Population%20Health%20Survey%202008-2009%20-%20February%202010.pdf archived at http://www.webcitation.org/6k8mCbGGY
NSW	2011	Sproston K, Hing N, Palankay C. Prevalence of gambling and problem gambling in New South Wales [Internet]. Ogilvy Illumination; 2012. Available from: https://www.liquorandgaming.justice.nsw.gov.au/Documents/gaming-and-wagering/problems-with-gambling/research/7.%20Prevalence%20of%20gambling%20and%20problem%20gambling%20in%20NSW%20-%20October%202012.pdf archived at http://www.webcitation.org/6k8mw0C4r
NT	1999	Productivity Commission. Australia's Gambling Industries. Report no.: 10.Canberra: Productivity Commission; 1999. Available from: http://www.pc.gov.au/inquiries/completed/gambling/report/gambling1.pdf archived at http://www.webcitation.org/6k8jP5l5E
NT	2005	Young M, Abu-Duhou I, Barnes T, Creed E, Morris M, Stevens M, et al. Northern Territory gambling prevalence survey 2005 [Internet]. Darwin: School for Social and Policy Research, Charles Darwin University; 2006. Available from: https://business.nt.gov.au/__data/assets/pdf_file/0010/275734/nt-gambling-prevalence-survey-2006.pdf archived at http://www.webcitation.org/6k8nEAPvy
QLD	1999	Productivity Commission. Australia's Gambling Industries. Report no.: 10.Canberra: Productivity Commission; 1999. Available from: http://www.pc.gov.au/inquiries/completed/gambling/report/gambling1.pdf archived at http://www.webcitation.org/6k8jP5l5E
QLD	2001	Department of Corrective Services, Queensland Government. Problem gambling prevalence survey 2002 [Internet]. Brisbane: Queensland Government; 2002. Available from: https://publications.qld.gov.au/storage/f/2014-06-20T02%3A36%3A55.113Z/problem-gambling-prevalence-survey-2002.pdf archived at http://www.webcitation.org/6k8ngnmfp
QLD	2003-04	Queensland Government. Queensland household gambling survey 2003-04 [Internet]. Brisbane: Queensland Government; 2007. Available from: https://publications.qld.gov.au/storage/f/2014-06-20T02%3A43%3A47.596Z/queensland-household-gambling-survey-2003-04.pdf archived at http://www.webcitation.org/6k9ukUDwQ
QLD	2006-07	Queensland Government. Queensland household gambling survey 2006-07 [Internet]. Brisbane: Queensland Government; 2008. Available from: https://publications.qld.gov.au/storage/f/2014-06-20T02%3A41%3A11.146Z/queensland-household-gambling-survey-2006-07.pdf archived at http://www.webcitation.org/6k9uw4x8N
QLD	2008-09	Queensland Government. Queensland household gambling survey 2008-09 [Internet]. Brisbane: Queensland Government; 2010. Available from: https://publications.qld.gov.au/storage/f/2014-06-20T02%3A40%3A21.071Z/queensland-household-gambling-survey-2008-09.pdf archived at http://www.webcitation.org/6k9v6PRqm
QLD	2011-12	Queensland Government. Queensland household gambling survey 2011-12 [Internet]. Brisbane: Queensland Government; 2012. Available from: https://publications.qld.gov.au/storage/f/2014-06-20T02%3A38%3A40.297Z/queensland-household-gambling-survey-2011-12.pdf archived at http://www.webcitation.org/6k9vFTo4d

SA	1996	Delfabbro P, Winefield AH. Community gambling patterns and the prevalence of gambling-related problems in South Australia (with particular reference to gaming machines). Adelaide: Department of Psychology, University of Adelaide; 1996.
SA	1999	Productivity Commission. Australia's Gambling Industries. Report no.: 10.Canberra: Productivity Commission; 1999. Available from: http://www.pc.gov.au/inquiries/completed/gambling/report/gambling1.pdf archived at http://www.webcitation.org/6k8jP5l5E
SA	2001	Taylor A, Dal Grande E, Gill T, Delfabbro P, Glenn V, Goulding S, et al. Gambling patterns of South Australians and associated health indicators [Internet]. Adelaide: South Australian Department of Human Services; 2001. Available from: https://health.adelaide.edu.au/pros/docs/reports/general/gambling_patterns_sa.pdf archived at http://www.webcitation.org/6k9wUxJXT
SA	2005	Taylor A, Gill T, Head L, Pugh L, Starr G, Rogers N, et al. Gambling prevalence in South Australia [Internet]. Adelaide: Department for Families and Communities, Government of South Australia; 2006. Available from: http://www.treasury.sa.gov.au/__data/assets/pdf_file/0015/1545/prevelance-study-report.pdf archived at http://www.webcitation.org/6k9wpKs9y
SA	2012	The Social Research Centre. Gambling prevalence in South Australia (2012) [Internet]. North Melbourne: The Social Research Centre; 2013. Available from: http://www.problemgambling.sa.gov.au/professionals/news_and_events/news-items/release-of-the-2012-gambling-prevalence-study-in-south-australia?a=13625 archived at http://www.webcitation.org/6k9x2DJAk
TAS	1994	Dickerson M, Walker M, Baron E. A baseline study of the extent and impact of gambling in Tasmania with particular reference to problem gambling. Sydney: Australian Institute for Gambling Research, University of Western Sydney; 1994.
TAS	1996	Dickerson M, Maddern R. The extent and impact of gambling in Tasmania with particular reference to problem gambling: A follow up to the baseline study conducted 1994. Sydney: Australian Institute for Gambling Research, University of Western Sydney; 1997.
TAS	1999	Productivity Commission. Australia's Gambling Industries. Report no.: 10.Canberra: Productivity Commission; 1999. Available from: http://www.pc.gov.au/inquiries/completed/gambling/report/gambling1.pdf archived at http://www.webcitation.org/6k8jP5l5E
TAS	2000	Roy Morgan Research. The third study into the extent and impact of gambling in Tasmania with particular reference to problem gambling : follow up to the baseline studies conducted in 1994 and 1996. Melbourne: Roy Morgan Research; 2001.
TAS	2005	Roy Morgan Research. The fourth study into the extent and impact of gambling in Tasmania with particular reference to problem gambling: Follow up to the studies conducted in 1994, 1996 and 2000. Melbourne: Roy Morgan Research; 2006.
TAS	2007	The SA Centre for Economic Studies. Social and economic impact study into gambling in Tasmania: Volume 2: The Prevalence Study [Internet]. Adelaide: University of Adelaide; 2008. Available from: http://www.treasury.tas.gov.au/domino/dtf/dtf.nsf/LookupFiles/Soc-Economic-Impact-Study-Vol2.pdf/$file/Soc-Economic-Impact-Study-Vol2.pdf archived at http://www.webcitation.org/6kA1g9Y8b
TAS	2011	The Allen Consulting Group, Problem Gambling Research and Treatment Centre, The Social Research Centre. Social and Economic Impact Study of Gambling in Tasmania: Volume 2: Gambling survey. Melbourne: Allen Consulting Group; 2011 p. http://www.treasury.tas.gov.au/domino/dtf/dtf.nsf/LookupFiles/Volume2secondgamblingSEIS.PDF/$file/Volume2secondgamblingSEIS.PDF archived at http://www.webcitation.org/6kA1Wrptl
TAS	2013	The Allen Consulting Group, The Social Research Centre, The Problem Gambling Research and Treatment Centre. Third social and economic impact

		study of gambling in Tasmania: Volume 2: 2013 Tasmanian gambling prevalence survey [Internet]. Melbourne: Allen Consulting Group; 2014. Available from: http://www.treasury.tas.gov.au/domino/dtf/dtf.nsf/LookupFiles/20150109SEISVolume2FINALREVISEDCHANGES.PDF/$file/20150109SEISVolume2FINALREVISEDCHANGES.PDF archived at http://www.webcitation.org/6kA1rNGoe
VIC	1997	Maddern C, Horman S, Dickerson M. Fifth community gambling patterns survey combined with second positive and negative perceptions of gambling survey. Melbourne: Victorian Casino and Gaming Authority; 1997.
VIC	1998	Roy Morgan Research. Sixth survey of community gambling patterns and perceptions [Internet]. Melbourne: Roy Morgan Research; 1999. Available from: http://assets.justice.vic.gov.au/vcglr/resources/9412cce8-63fe-4f5e-82e0-fe5c0951b62a/6thsurveycommunitygamblingpattersnandperceptions.pdf archived at http://www.webcitation.org/6kA2LeY36
VIC	1999a	Roy Morgan Research. Seventh survey of community gambling patterns and perceptions [Internet]. Melbourne: Roy Morgan Research; 2000. Available from: http://assets.justice.vic.gov.au/vcglr/resources/4ea71095-8ecc-44e4-a096-5477dfaf4f06/7thsurveycommunitygamblingpatternsandperceptions.pdf archived at http://www.webcitation.org/6kA2dQikr
VIC	1999b	Productivity Commission. Australia's Gambling Industries. Report no.: 10.Canberra: Productivity Commission; 1999. Available from: http://www.pc.gov.au/inquiries/completed/gambling/report/gambling1.pdf archived at http://www.webcitation.org/6k8jP5l5E
VIC	2003	McMillen J, Marshall D, Ahmed E, Wenzel M. 2003 Victorian Longitudinal Community Attitudes Survey [Internet]. Canberra: The Centre for Gambling Research, The Australian National University; 2004. Available from: http://hdl.handle.net/1885/45189 archived at http://www.webcitation.org/6kA31QkOI
VIC	2007	Thomas SA, Jackson AC. Risk and protective factors, depression and comorbidities in problem gambling. Melbourne: beyondblue; 2008.
VIC	2008	Hare S. A study of gambling in Victoria: Problem gambling from a public health perspective [Internet]. Melbourne: Department of Justice, Government of Victoria; 2009. Available from: https://www.responsiblegambling.vic.gov.au/__data/assets/pdf_file/0013/4027/A-study-of-gambling-in-Victoria-PRINT-Sept-10.pdf archived at http://www.webcitation.org/6kA3s64IZ
VIC	2014	Hare S. Study of gambling and health in Victoria: Findings from the Victorian prevalence study 2014 [Internet]. Melbourne: Victorian Responsible Gambling Foundation; 2015. Available from: https://www.responsiblegambling.vic.gov.au/__data/assets/pdf_file/0018/25551/Study_of_gambling_and_health_in_Victoria.pdf archived at http://www.webcitation.org/6kA43TuMs
WA	1994	Dickerson M, O'Connor J, Baron E. An assessment of the extent and degree of gambling related problems in the population of Western Australia. Sydney: Australian Institute for Gambling Research, University of Western Sydney; 1994.
WA	1999	Productivity Commission. Australia's Gambling Industries. Report no.: 10.Canberra: Productivity Commission; 1999. Available from: http://www.pc.gov.au/inquiries/completed/gambling/report/gambling1.pdf archived at http://www.webcitation.org/6k8jP5l5E
